# Frailty, cognitive impairment, and depressive symptoms in Chinese older adults: an eight-year multi-trajectory analysis

**DOI:** 10.1186/s12877-023-04554-1

**Published:** 2023-12-12

**Authors:** Yiyang Yuan, Changmin Peng, Jeffrey A. Burr, Kate L. Lapane

**Affiliations:** 1 Department of Population and Quantitative Health Sciences, University of Massachusetts Chan Medical School, 368 Plantation Street, 01605 Worcester, MA USA; 2 Department of Gerontology, University of Massachusetts Boston, Boston, MA USA

**Keywords:** CHARLS, Multi-trajectory model, Physical frailty, Cognitive health, Mental health, Human capital, Social capital, Financial capital, Health capital

## Abstract

**Background:**

Frailty, cognitive impairment, and depressive symptoms are closely interrelated conditions in the aging population. However, limited research has longitudinally analyzed the concurrent trajectories of these three prominent conditions in older adults in China. This study aimed to explore the eight-year trajectories of frailty, cognitive impairment, and depressive symptoms, and to identify individual-level and structural-level factors associated with the trajectories.

**Methods:**

Four waves of data from the *China Health and Retirement Longitudinal Study* (2011–2018) were used to identify 6,106 eligible older adults. The main measures included frailty by the frailty index constructed using 30 indicators, cognitive impairment by the summary score of immediate and delayed word recall, figure drawing, serial subtraction, and orientation, and depressive symptoms by the Center for Epidemiologic Studies Depression Scale. Multi-trajectory models identified the trajectories of frailty, cognitive impairment, and depressive symptoms over time. Multinomial logistic regression was employed to estimate the associations between individual-level capital factors and one structural factor (*hukou* and geographic residency) with the identified trajectories, adjusting for demographic characteristics.

**Results:**

Four trajectories emerged: (1) worsening frailty, worsening cognitive impairment, depression (14.0%); (2) declining pre-frailty, declining cognition, borderline depression (20.0%); (3) pre-frailty, worsening cognitive impairment, no depression (29.3%); and (4) physically robust, declining cognition, no depression (36.7%). Using the “physically robust, declining cognition, no depression” as the reference, not working, no social activity participant, worse childhood family financial situation, and poorer adult health were most strongly associated with the “worsening frailty, worsening cognitive impairment, depression” trajectory; worse health during childhood had the highest association with the “declining pre-frailty, declining cognition, borderline depression” trajectory; less education, lower household consumption, and rural *hukou* had the greatest association with the increased likelihood of the “pre-frailty, worsening cognitive impairment, no depression” trajectory.

**Conclusions:**

Findings could inform the understanding of the interrelationship of frailty, cognitive impairment, and depressive symptoms in older adults in China and may help practitioners detect adults at risk for adverse trajectories to implement strategies for proper care.

**Supplementary Information:**

The online version contains supplementary material available at 10.1186/s12877-023-04554-1.

## Introduction

The older adult population in China nearly doubled in the past decade, from 127.8 million in 2012 to 200.6 million in 2021 [1]. This population often experienced frailty, with estimates from 7 to 26% [2, 3]. In those aged ≥ 60 years, over 15% had mild cognitive impairment, and 6.0% and 3.9% had dementia and Alzheimer’s disease, respectively [4, 5]. Depressive symptoms are present in 44.5% of older adults in China [6]. With the rapid growth of this aging population, understanding how frailty, cognitive impairment, and depressive symptoms progress jointly over time and what factors contribute to these trajectories is vital for facilitating interventions to help manage these conditions and promote healthy aging.

Prior studies highlighted the interrelationships among frailty, cognitive impairment, and depressive symptom among older adults living in China. More severe depressive symptoms were associated with a higher level of frailty [7], and frailty predicted incident depressive symptoms [8]. The one-year incidence of frailty is estimated to be 19.5% higher in older adults with cognitive impairment compared to those without cognitive impairment [9]. Chinese older adults with depressive symptoms had worse cognitive performance and were more prone to mild cognitive impairment than their counterparts without depressive symptoms [10, 11]. However, most studies were either based on cross-sectional designs or only addressed two of these three closely related conditions. Research that simultaneously examined the longitudinal progression of frailty, cognitive impairment, and depressive symptoms in older adults in China is critical to achieving a more holistic understanding of how these conditions relate to one another.

The severity and/or rate of how frailty, cognitive impairment, and depressive symptoms progress longitudinally may vary from one subgroup of older adults to another [12–14]. Furthermore, relatively worse trajectories of physical frailty were associated with greater odds of worse trajectories of cognitive impairment [15, 16], and older adults who exhibited trajectories of few or decreasing depressive symptoms were less likely to experience trajectories of poor cognition [17]. Findings from these studies suggest the heterogeneity of and the association between the progression of frailty, cognitive impairment, and depressive symptoms. The present study seeks to extend this line of work by identifying the concurrent trajectories of frailty, cognitive impairment, and depressive symptoms and to describe the extent to which these trajectories vary among Chinese older adults.

Many risk factors have been identified as being associated with frailty, cognitive impairment, and depressive symptoms. These risk factors can be found at the individual level, including demographic characteristics, as well as factors associated with health capital, human capital, social capital, and financial capital [18–24]. Other risk factors are located at the structural level. Among Chinese older adults, individual-level health capital indicators, such as health status during childhood and adulthood, human capital indicators, such as educational attainment, social capital indicators, such as living with a partner and adult children and social activity participation, financial capital indicators, such as household income, and structural factors, such as residing in urban areas with rural *hukou* status (Chinese household registration system), have been found to be either positively or negatively associated with frailty, cognitive impairment, and depressive symptoms [3, 6, 25–30]. Building on previous research, the present study contributed to the scientific literature by examining how these risk factors were associated with the concurrent trajectories of frailty, cognitive impairment, and depressive symptoms.

The objectives of this longitudinal study were to identify the concurrent trajectories of frailty, cognitive function, and depressive symptoms in a national cohort of older adults in China, and to quantify the impact of individual-level factors of human, social, financial and health capitals and structural factor for the identified trajectories. We hypothesized that the concurrent trajectories of frailty, cognitive impairment, and depressive symptoms would be heterogeneous, and that Chinese older adults with better individual-level capital and structural factors would have less detrimental trajectories.

## Methods

### Data and sample

We used data from the *China Health and Retirement Longitudinal Study* (CHARLS), which was designed based on the *U.S. Health and Retirement Stud*y. Trained interviewers conducted in-person interviews with Chinese respondents aged ≥ 45 years to collect nationally representative data on demographic characteristics, family structure, social engagement, physical, cognitive, and psychosocial well-being, work history and pensions, and economic standing [31]. We used four waves of the CHARLS core survey [2011 (baseline); 2013; 2015; 2018], along with the Life History survey (2014) that included all living respondents from the first two waves. To ensure data accessibility and comparability, we used the Harmonized CHARLS Version D curated by the USC Gateway to Global Aging Data [32] linked to the original CHARLS to obtain variables not included in the Harmonized dataset, but essential to this analysis.

In the main analysis, respondents were included if they participated in the baseline wave and at least one other wave, aged ≥ 60 years at baseline, and had valid information for frailty, cognition, and depressive symptoms for at least two waves. The sample size was 6,106 (Supplemental Fig. 1). For sensitivity analysis, we further restricted respondents to those with at least three waves of valid data for the three conditions, resulting in a sample size of 5,182 (Supplemental Fig. 2).

### Outcome measures

*Frailty* was measured with a frailty index (FI), constructed from 30 indicators, including thirteen health-related diagnoses from physicians, five on disabilities, and twelve on limitations with activities of daily living (ADLs) and instrumental activities of daily living (IADLs) (see Supplemental Table 1). The indicators were selected following recommended procedures [33] and scored consistent with previous studies [7, 34–39]. The sum of the individual indicator scores was divided by 30 to create the FI (range: 0–1). Higher scores indicate greater frailty. Thresholds of 0.1 and 0.2 for physically robust (FI ≤ 0.10), pre-frailty (0.1 < FI ≤ 0.2), and frailty (FI > 0.2) were used as guidelines to interpret the identified trajectories [35].

*Cognitive function* was measured with (1) episodic memory (range: 0–10), using the average score of immediate word recall (range: 0–10) and delayed word recall (range: 0–10), (2) orientation and attention, using items from Telephone Interview of Cognitive Status (TICS-10) on naming of date, day of the week, and season (range: 0–5) and serial 7’s (range: 0–5), and (3) visuoconstruction, using figure drawing (range: 0–1). Scores from each assessment were summed to create an overall cognitive function score (range: 0–21) [40–45]. Lower scores indicated lower cognitive function.

*Depressive symptoms* were measured by the summary score from the validated Chinese version of the 10-item Center for Epidemiologic Studies Depression Scale (CES-D; score: 0–30). Higher scores indicate more depressive symptoms. A threshold of 12, which indicated possible clinical depression, was used to guide the interpretation of the identified trajectories [46].

Frailty, cognition, depressive symptoms were measured in the same way across four waves (Supplemental Table 2).

### Individual-level demographics, capital factors and structural factor

Demographic characteristics included age (in years) and sex (1 = *female*; 2 = *male*, reference).

Human capital included education level (1 = *no formal education*; 2 = *less than elementary school*; 3 = *elementary school or higher*, reference); and work status (1 = *currently working*, reference; 2 = *retired*; 3 = *not employed/never worked*).

Social capital included marital status (1 = *married*, reference; 2 = *not married*, including widowed, divorced, separated, or never married); living arrangements with children (1 = *living with children*, reference; 2 = *empty nester*, i.e., not living with children); and social activity participation, including interacting with friends, playing Ma-Jong/chess/cards or going to community/sport/social or other clubs, taking part in a community-related organization, doing voluntary or charity work, and attending an educational or training course (1 = *any social activity participation*, reference; 2 = *no participation in any social activities*).

Financial capital included respondents’ self-rated childhood family financial situation before aged 17 years, compared to the average family in the same community or village (1 = *a lot/somewhat better off*; 2 = *the same*, reference; 3 = *somewhat/a lot worse off*); and adulthood financial situation by *per capita* household consumption (in Chinese Yuan) at baseline in quartiles.

Health capital included respondents’ self-rated childhood health before age 16 years, compared to other children of the same age (1 = *much/somewhat healthier*, 2 = *about average*, reference; 3 = *somewhat less/much healthier*; and adulthood health self-reported at baseline (1 = *very good/good*, reference; 2 = *fair*; 3 = *poor/very poor*).

One structural factor included *hukou* status and residence, a composite measure combining *hukou* status and residence classification, providing a fuller representation of rural-urban inequalities (1 = rural residence with rural *hukou*; 2 = rural residence with urban *hukou*; 3 = urban residence with rural *hukou*; 4 = urban residence with urban *hukou*, reference) Of note, *hukou*, the unique Chinese government household registration system, is profoundly tied with numerous aspects of a person’s life chances, including education, healthcare, housing, and social welfare [47]. Research shows the intersection of *hukou* status and rural/urban residence is associated with a variety of health outcomes, where those with rural *hukou* and rural residence represent the most disadvantaged group [48].

All variables were time-invariant and measured at baseline.

### Analytic Strategy

Descriptive statistics for baseline characteristics are presented, with median and interquartile range (IQR) reported for non-normally distributed continuous variables and percentages reported for categorical variables. Multi-trajectory modeling was used to identify the concurrent trajectories of frailty, cognitive impairment, and depressive symptoms. This modeling technique defines each trajectory group as a set of trajectories for multiple interrelated outcomes [49]. In the context of this study, each trajectory group consisted of one trajectory for frailty, one trajectory for cognitive impairment, and one trajectory for depressive symptoms.

The first step was to determine the number of trajectories that best fitted the data. Individual trajectories of frailty, cognitive impairment, and depressive symptoms were initially assumed to take a cubic shape, the highest possible order with four time points, and multi-trajectory models with two to six trajectory groups were estimated. To examine model fit, we compared the Bayesian Information Criterion (BIC; models with higher BIC scores are preferred), the group average posterior probability of assignment (AvePP; an AvePP > 0.7 for all trajectory groups indicated acceptable group assignment certainty), and the odds of correct classification (OCC; an OCC > 5 for all trajectory groups indicated high assignment accuracy). We also evaluated graphic depictions of each model to see if increasing the number of trajectory groups would identify a new trajectory of frailty, cognitive impairment, and depressive symptoms or overlap with the existing ones. With the optimal number of trajectories determined, the next step was to adjust the shape parameters to improve model fit. If the highest order of the shape parameters was not statistically significant (*p*-value ≥ 0.05), the parameters were reduced one step at a time from cubic to quadratic and then from quadratic to linear, as necessary, until the highest order of the shape parameters of all trajectories were significant. The model that best fit the data was thus identified [49, 50].

The identified trajectories of frailty, cognitive impairment, and depressive symptoms were graphically represented using solid lines for the trajectories based on model predicted scores and dashed lines for the 95% confidence interval (CI) bands. We reported the percentage of respondents belonging to each group and described each trajectory with qualitative labels. Respondents were then assigned to the trajectory group to which they had the highest posterior probability of belonging. Multinomial logistic models were used to estimate the association between human, social, financial, health capital factors and structural factors and the trajectory groups, adjusting for demographic characteristics. Correlations among the baseline characteristics were examined to ensure minimal multicollinearity. Results were presented as adjusted odds ratios (aOR) with 95% CIs. *P*-values have been corrected for multiple comparison using the Holm step-down approach [51].

The same analytical procedure was carried out for the sensitivity analysis sample. Multi-trajectory models were fit using *traj* package in Stata 15 [52]. Multinomial logistic models were estimated in Mplus 8.4 [53] with full information maximum likelihood method to account for missing values in the covariates. All other analyses were conducted in SAS 9.4 [54]. Figures were created in R with *ggplot2* [55].

## Results

### Sample characteristics

As shown in Table 1, at baseline, the median age of the study sample was 66.0 years (IQR: 9.0) and half were female. Over one-third had no formal education, and most were either currently working (49.7%) or retired (47.9%). One in five respondents were widowed, divorced, separated, or never married, while over half were empty nesters. Over half reported no participation in any social activities. About 41% reported their childhood family financial situation was somewhat or a lot worse than average in the same community or village. The median baseline household annual *per capita* consumption was about 4,459 Chinese Yuan (interquartile range: 5,511). Over half of respondents rated their childhood health as about average compared to others of the same age, and 12.6% rated as poor health than their contemporaries. While one-half of respondents rated their baseline health to be fair, almost one-third rated it as poor or very poor. About 59% resided in rural areas with rural *hukou.*

**Table 1:** Baseline characteristics by the trajectories of frailty, cognitive impairment, and depressive symptoms in older adults in China (2011–2018)

		All
		(n = 6,106)
**Age** (median, IQR)		66.0 (9.0)
**Female** (%)		49.7
***Human capital***		
**Education** (%)		
	No formal education	35.2
	Less than elementary school	20.8
	Elementary school or higher	44.0
**Work status** (%)		
	Currently working	49.7
	Retired	47.9
	Not employed/Never worked	2.5
***Social capital***		
Not married ^a^ (%)		19.8
Empty nester (%)		54.0
No participation in any social activities (%)		54.8
***Financial capital***		
**Childhood family financial situation**^b^ (%)		
	A lot/somewhat better off	9.0
	Same	50.1
	Somewhat/A lot worse off	40.9
**Per capita household consumption at baseline** (in Chinese Yuan; %)		
	Quartile 1 [ 0.0–2638.0)	25.0
	Quartile 2 [2638.0–4458.8)	25.0
	Quartile 3 [4458.8–8149.0)	25.0
	Quartile 4 [8149.0–222,640]	25.0
***Health capital***		
**Childhood health**^c^ (%)		
	Much/somewhat healthier	34.6
	About average	52.8
	Somewhat less healthy/Much less healthy	12.6
**Self-rated health at baseline** (%)		
	Very good/good	19.4
	Fair	49.3
	Poor/very poor	31.4
***Structural factor***		
***Hukou*and residence** (%)		
	Rural *hukou*, rural residence	59.2
	Rural *hukou*, urban residence	17.5
	Urban *hukou*, rural residence	2.9
	Urban *hukou*, urban residence	20.4

Notes. IQR = interquartile range

^a^ Including widowed, divorced, separated, and never married

^b^ Self-rated family financial situation before age 17 years, compared to the average family in the same community/village

^c^ Self-rated health before age 16 years, compared to other children of the same age

### Trajectories of frailty, cognitive impairment, and depressive symptoms

**Fig. 1:**
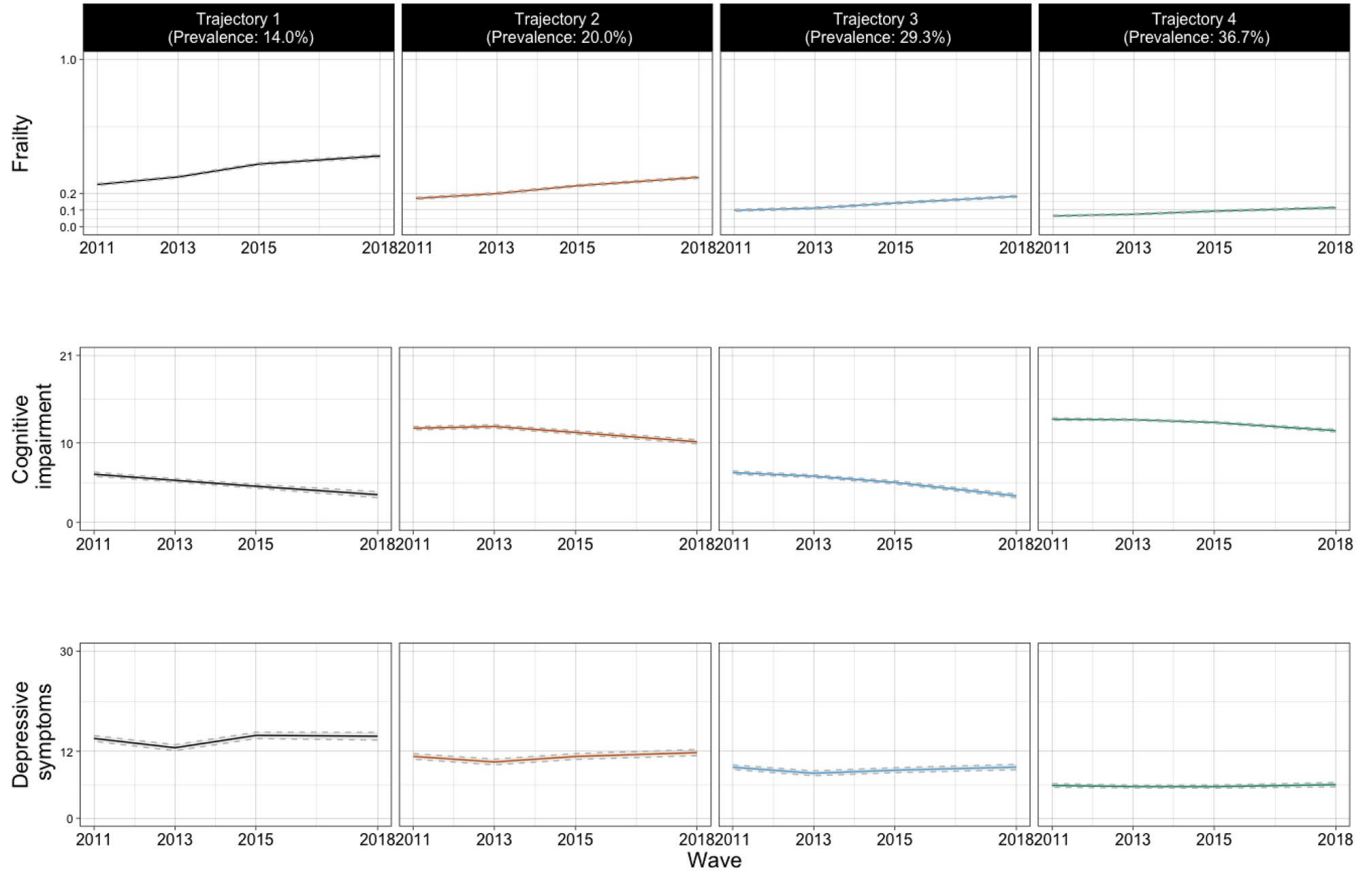
Trajectories of frailty, cognitive impairment, and depressive symptoms in older adults in China (2011–2018). Notes. Trajectory 1 = Worsening frailty, worsening cognitive impairment, depression; Trajectory 2 = Declining pre-frailty, declining cognition, borderline depression; Trajectory 3 = Pre-frailty, worsening cognitive impairment, no depression; Trajectory 4 = Physically robust, declining cognition, no depression

The best fitting multi-trajectory model identified four trajectories of frailty, cognitive impairment, and depressive symptoms (Supplemental Table 3), which were presented in Fig. 1 with parameter estimates in Supplemental Table 4. Respondents belonging to the first trajectory (“worsening frailty, worsening cognitive impairment, depression;” prevalence: 14.0%) were characterized as being frail, cognitively impaired, and depressed at baseline. Their frailty and impaired cognition continued to worsen over time, and their depressive symptoms appeared to be relatively stable, above the clinical threshold. Respondents belonging to the second trajectory (“declining pre-frailty, declining cognition, borderline depression;” prevalence: 20.0%) were pre-frail at baseline and became frail over time. Their cognition at baseline was better than those in the first trajectory; while it did decline, by the end of the follow-up, these respondents appeared to have better cognitive function than those in the first trajectory. They had borderline depression at baseline, with few changes throughout the observation period. Respondents belonging to the third trajectory (“pre-frailty, worsening cognitive impairment, no depression;” prevalence: 29.3%) remained pre-frail over time, with a trajectory of worsening cognitive impairment similar to the first trajectory group, and their depressive symptoms were below the clinical threshold during the follow-up period. Respondents belonging to the fourth trajectory (“physically robust, declining cognition, no depression;” prevalence: 36.7%) were not frail, had higher baseline cognitive functioning and experienced declining cognitive functioning over time, similar to those in the second trajectory, with depressive symptoms below the clinical threshold during follow-up.

### Predictors of the trajectories

Respondents were assigned to the trajectory to which they had the highest posterior probability of belonging (Supplemental Table 5). Table 2 shows the associations between the individual-level capital and structural factors and the identified trajectories adjusting for respondents’ age and sex, using the comparatively healthier “physically robust, declining cognition, no depression” trajectory as the reference, with *P*-values corrected for multiple comparison summarized in Supplemental Table 6.

*Human capital*. Respondents that received no formal education were 13 times as likely to experience “worsening frailty, worsening cognitive impairment, depression” [aOR: 12.99; 95%CI: 9.84–17.15] and 23 times as likely to experience “pre-frailty, worsening cognitive impairment, no depression” [aOR: 23.14; 95%CI: 18.43–29.07]. Retirees [aOR: 2.66; 95%CI: 2.08–3.40] and respondents who were not employed or had never worked [aOR: 3.05; 95%CI: 1.57–5.92] were most likely to experience “worsening frailty, worsening cognitive impairment, depression,” and significantly more likely to experience “declining pre-frailty, declining cognition, borderline depression.”

*Social capital*. Not being married and living without children in the household did not appear to be significantly associated with any of the trajectory groups, after the correction for multiple comparisons. Respondents with no social activity participation were nearly twice as likely to experience “worsening frailty, worsening cognitive impairment, depression” [aOR: 1.98; 95%CI: 1.61–2.45] and “pre-frailty, worsening cognitive impairment, no depression” [aOR: 1.69; 95%CI: 1.43–2.01].

*Financial capital*. Older adults reporting worse childhood family financial situations were 66% more likely to experience “worsening frailty, worsening cognitive impairment, depression” [aOR: 1.66; 95%CI: 1.33–2.07]. Being in the bottom two quartiles of annual *per capita* household consumption was associated with a greater likelihood of being in the “pre-frailty, worsening cognitive impairment, no depression” trajectory.

**Table 2:** Association between baseline characteristics and the trajectories of frailty, cognitive impairment, and depressive symptoms in older adults in China (2011–2018)

		Trajectories of frailty, cognitive impairment, and depressive symptoms											
		(*Ref: Physically robust, declining cognition, no depression*)											
		Worsening frailty, worsening cognitive impairment, depression			Declining pre-frailty, declining cognition, borderline depression				Pre-frailty, worsening cognitive impairment, no depression				
		aOR	95% CI		aOR	95% CI			aOR	95% CI			
**Age** (every 5-year increase; continuous)		1.93*	(1.77–2.13)		1.16*	(1.07–1.26)			1.68*	(1.55–1.83)			
**Female** (ref: Male)		2.66*	(2.11–3.36)		1.67*	(1.41–1.98)			2.31*	(1.93–2.77)			
***Human capital***													
**Education** (ref: Elementary school or higher)													
	No formal education	12.99*	(9.84–17.15)		1.37	(1.06–1.76)			23.14*	(18.43–29.07)			
	Less than elementary school	2.43*	(1.83–3.24)		1.39*	(1.14–1.69)			4.31*	(3.48–5.35)			
**Work status** (ref: Currently working)													
	Retired	2.66*	(2.08–3.40)		1.49*	(1.23–1.80)			1.12	(0.92–1.37)			
	Not employed/Never worked	3.05*	(1.57–5.92)		2.19*	(1.31–3.66)			1.17	(0.63–2.17)			
***Social capital***													
**Not married**^**a**^ (ref: Married)		1.39	(1.07–1.80)		1.22	(0.98–1.51)			1.33	(1.06–1.66)			
**Empty nester** (ref: Living with children)		0.88	(0.71–1.08)		0.99	(0.84–1.16)			0.89	(0.75–1.06)			
**No participation in any social activities** (ref: Any social activity participation)		1.98*	(1.61–2.45)		1.26	(1.07–1.47)			1.69*	(1.43–2.01)			
***Financial capital***													
**Childhood family financial situation**^**b**^ (ref: Same)													
	A lot better off/somewhat better off	1.28	(0.85–1.93)		1.23	(0.93–1.64)			1.22	(0.90–1.67)			
	Somewhat worse off/A lot worse off	1.66*	(1.33–2.07)		1.28	(1.08–1.52)			1.15	(0.96–1.38)			
**Per capita household consumption** (ref: Quartile 4)													
	Quartile 1	1.18	(0.83–1.66)		0.97	(0.74–1.27)			1.58*	(1.19–2.10)			
	Quartile 2	1.28	(0.91–1.78)		1.06	(0.83–1.37)			1.65*	(1.26–2.18)			
	Quartile 3	1.08	(0.78–1.50)		1.17	(0.93–1.47)			1.27	(0.97–1.68)			
***Health capital***													
**Childhood health**^**c**^ (ref: About average)													
	Much healthier/somewhat healthier	0.75	(0.59–0.95)		0.85	(0.72–1.02)			0.75*	(0.62–0.90)			
	Somewhat less healthy/Much less healthy	1.34	(0.97–1.84)		1.47	(1.13–1.91)			1.12	(0.84–1.48)			
**Self-rated health** (ref: Fair)													
	Very good/good	0.32*	(0.23–0.46)		0.31*	(0.24–0.40)			0.60*	(0.48–0.74)			
	Poor/very poor	9.76*	(7.69–12.39)		4.59*	(3.83–5.51)			2.23*	(1.81–2.75)			
***Structural factor***													
***Hukou*and residence** (ref: Urban *hukou*, urban residence)													
	Rural *hukou*, rural residence	3.98*	(2.83–5.61)		1.40	(1.11–1.75)			4.21*	(3.17–5.60)			
	Rural *hukou*, urban residence	2.02*	(1.38–2.95)		0.92	(0.71–1.19)			2.65*	(1.94–3.63)			
	Urban *hukou*, rural residence	1.58	(0.75–3.35)		1.13	(0.73–1.74)			1.71	(0.97–3.01)			

Notes. Ref = reference. aOR = adjusted odds ratio. 95% CI = 95% confidence interval

* *P*-value adjusted for multiple comparisons (Holm step-down method) < 0.05. For specific *P*-values, both original and adjusted for multiple comparisons, see Supplemental Table 6.

^a^ Included widowed, divorced, separated, and never married.

^b^ Self-rated family financial situation before age 17 years, compared to average family in the same community/village.

^c^ Self-rated health before age 16 years compared to other children of the same age.

*Health capital.* After correction for multiple comparison, better health during childhood was only significantly associated with lower likelihood of belonging to the “pre-frailty, worsening cognitive impairment, no depression” trajectory. Older adults who rated themselves as in very good or good health at baseline were 40–69% less likely to be in one of these three trajectories, while those with poor or very poor health were about 2.2 to 9.8 times as likely to do so.

*Structural factor*. Respondents living in rural areas with rural *hukou* were four times as likely, and those in rural areas with urban *hukou* were over twice as likely to belong to the “worsening frailty, worsening cognitive impairment, depression” [aOR_rural *hukou*, rural residence_: 3.98; 95%CI: 2.83–5.61; aOR_rural *hukou*, urban residence_: 2.02; 95%CI: 1.38–2.95] and the “pre-frailty, worsening cognitive impairment, no depression” [aOR_rural *hukou*, rural residence_: 4.21; 95%CI: 3.17–5.60; aOR_rural *hukou*, urban residence_: 2.65; 95%CI: 1.94–3.63] trajectories, respectively.

### Sensitivity analysis

Comparing the main and the sensitivity analysis sample which required at least three waves of data, there were minimal differences in baseline characteristics and the distribution of frailty, cognition, and depressive symptoms across waves (Supplemental Tables 7–9). The best-fitted multi-trajectory model indicated four identical trajectories with slightly different shape parameters (Supplemental Fig. 3; Supplemental Tables 10–11). In the multinomial model, the association between individual-level capital factors and structural factor and the identified trajectories were consistent with the main analysis (Supplemental Tables 12–13). The only exception was that the associations between work status and the identified trajectories in the sensitivity analysis sample were smaller, which likely resulted from the difference in the distribution of this variable with regards to the trajectories between the two samples (Supplemental Tables 5 & 7).

## Discussion

In a cohort of Chinese older adults followed up for eight years, we identified four distinctive trajectories of frailty, cognitive impairment, and depressive symptoms. We also demonstrated that multiple individual-level characteristics and one structural factor were associated with experiencing these trajectories. This study offered several important innovations relative to previously published research in this field. First, despite acknowledging the connections of frailty, cognitive impairment, and depressive symptoms, prior research did not analyze all three conditions as outcomes simultaneously. This is important because not doing so overlooks the fact that health is a holistic concept that encompass multiple dimensions of well-being, including physical, cognitive, and mental health. Previous efforts focusing on frailty and cognitive impairment proposed the construct of “cognitive frailty” [56], but this construct has been inconsistently operationalized and, in some cases, the operationalization may have been too general (i.e., identifying four groups from a cross-tabulation of the presence or absence of frailty and cognitive impairment) to shed light on the intricate relationships between the progression of these conditions [25]. In contrast, we measured frailty, cognition, and depressive symptoms as individual constructs using well-established instruments and we employed a longitudinal study design with a multi-trajectory modeling approach to evaluate how these conditions progress concurrently.

Importantly, the finding of four types of trajectories indicates that the progression of frailty, cognitive impairment, and depressive symptoms in Chinese older adults is heterogeneous. One recent study analyzed the first three waves of CHARLS data using a mixed effects generalized linear model and found that impairments in physical functioning (mobility, ADLs, and IADLs) increased over time, and that increasing impairment rates were found among older adults with greater depressive symptoms but similar across cognitive function levels [57]. While our study focused on frailty rather than physical functioning impairment, the operationalization of these two constructs overlapped with respect to limitations in ADLs and IADLs. To this degree, our findings are consistent with this previous work, as the progression of frailty was more apparent in older Chinese adults with a higher number of depressive symptoms – older Chinese adults in the “worsening frailty, worsening cognitive impairment, depression” trajectory experienced increasing frailty over time and possible clinical depression, while those belonging to the “physically robust, declining cognition, no depression” trajectory remained below the respective clinical thresholds for frailty and depressive symptoms. More importantly, our findings added to this earlier research by showing that the rate of increased frailty may not be the same for all older Chinese adults, as they may follow distinct trajectories. Notably, the progression of frailty appeared to vary among our sample of older Chinese adults who experienced a similar progression of cognitive impairment – older Chinese adults in the “worsening frailty, worsening cognitive impairment, depression” and those in the “pre-frailty, worsening cognitive impairment, no depression” trajectories had a similar progression in terms of cognitive impairment, but a different progression of frailty. Taken together, the heterogeneity captured in our study indicated that the relationships between these conditions differ from one subpopulation of older adults to another, hence marginal estimates of the associations provided in earlier studies may not be adequate. Quantifying how these conditions are associated within the subpopulations is beyond the scope of the current study, as multi-trajectory modeling was not designed to quantify the actual associations, but this issue should be evaluated in future work where data are available, and methods allow for the estimation of these relationships.

Our findings can also be informative for healthcare practice because older Chinese adults may benefit from care tailored to their specific progression profiles rather than applying a “one-size-fits-all” approach. Ideally, in the presence of these interrelated conditions, comprehensive care should be provided to mitigate the progression of these conditions. However, availability of community-based long-term care is scarce in China, especially in rural communities [58], where older adults had substantially higher risks for adverse trajectories. As such, it is crucial to identify and triage older adults at greater risk for worse trajectories, with care tailored to their specific profiles of frailty, cognitive impairment, and depressive symptoms. To aid the identification of older adults at risk for adverse multi-condition trajectories, we distilled previous published work on the characteristics associated with frailty, cognitive impairment, and depressive symptoms in Chinese older adults into factors on the individual level reflecting human capital, social capital, financial capital, and health capital as well as on the structural level to explore a wide range of risk factors. Our results indicated that not currently working, lack of social activity participation, worse childhood family financial situation, and poor self-rated health at baseline were the strongest predictors for the “worsening frailty, worsening cognitive impairment, depression” trajectory. Worse childhood health was most strongly related with the “declining pre-frailty, declining cognition, borderline depression” trajectory. Less education, lower per capita household consumption, and having a having a rural *hukou* had the highest association with the “pre-frailty, worsening cognitive impairment, no depression” trajectory. Additionally, some factors can serve as modifiable intervention targets. For example, more social participation both in terms of frequency and variety has been shown to have protective effects over from frailty, cognitive impairment, and depressive symptoms among Chinese older adults [59–61]. Given the association between social participation and the identified trajectories, engaging at-risk older adults in proper social activities may help alleviate the progression of these conditions.

Our results may not directly comparable with previous work, because to the best of our knowledge, this was the first study that examined the concurrent trajectories of frailty, cognitive impairment, and depressive symptoms as the outcome. But the findings were consistent with prior work in terms of the association of various capital factors, rural/urban residence and *hukou* status with frailty, cognitive impairment, and depressive symptoms as individual outcomes [3, 6, 25–30]. Notably, lack of formal education appeared to be the driving force for trajectories marked by worsening cognitive impairment. This was expected given that it has been established association between higher education attainment and better cognitive function in late life [62]. But the magnitude of this association shown in this study was alarming. After correction for multiple comparison, childhood health and financial situation did not appear to be a significant risk factor for the comparatively worse trajectories, although previous literature has indicated the lifelong impact of childhood adversities on these three conditions in later life [63–65]. This could also be due to the observed strong association between participants’ self-rated health at baseline and the trajectories. Future work may consider further quantify how much of the effect on the trajectories of frailty, cognitive impairment, and depressive symptoms at later life would be mediated by the baseline health. More importantly, our findings underscore the urgency of engaging older adults in activities beneficial for cognitive function [66] to reduce the rate of further cognitive decline, as we cannot go back in time to change older adult’s education attainment or adverse childhood circumstances.

We note some limitations. Older adults who were lost-to-follow-up early on during the CHARLS survey, or were too physically frail, cognitively impaired, and/or depressed to complete the assessments were excluded, so selection bias cannot be ruled out. Missing valid measures on the three conditions for more than one wave may be of concern, but the sensitivity analysis of participants who had valid measures for at least three waves gave confidence regarding the robustness of our results. The length of follow-up was up to eight years, which may not be enough to observe substantial changes in frailty, cognitive impairment, and depressive symptoms [67–69]. Human, social, financial, and health capital factors were assessed at baseline, but some of these may change over time. If and to what extent the older adults’ trajectories would be further impacted by these changes in these capital factors should be examined in future studies. Due to data limitations, we did not assess factors associated with other types of capital factor such as “cultural capital” [70], nor other clinical characteristics that may influence the progression of the three conditions. Due to model constraints, we could not incorporate population sample weights in the multi-trajectory model. While the data collected in CHARLS were designed to be nationally representative, the findings may not be generalizable to the entire Chinese older adult population.

## Conclusions

We contributed to the literature by identifying four trajectories of frailty, cognitive impairment, and depressive symptoms in the rapidly growing Chinese older adult population over a span of eight years. Notably, more than one-in-seven experienced worsening in frailty and cognition with persistent depression, and nearly one-in-three experienced pre-frailty and worsening in cognition without depression. The identified trajectories shed light on the heterogeneity in the progression of and interrelationship among frailty, cognitive impairment, and depressive symptoms. The human, social, financial and health capital factors and the structural factor found to be associated with the trajectories provided insights for identifying at-risk populations and giving impetus for the implementation of proper care to prevent more detrimental progression of these prominent aging-related conditions.

### Electronic supplementary material

Below is the link to the electronic supplementary material.

Supplementary Material 1

## Data Availability

This analysis uses the Harmonized CHARLS dataset and Codebook, Version D as of June 2021 developed by the Gateway to Global Aging Data, with linkage to the original CHARLS data to obtain variables not included in the Harmonized CHARLS Version D. The development of the Harmonized CHARLS was funded by the National Institute on Aging (R01 AG030153, RC2 AG036619, R03 AG043052). For data access and more information, please refer to https://g2aging.org/.
