# Supplementary material for: Frailty, cognitive impairment, and depressive symptoms in Chinese older adults: an eight-year multi-trajectory analysis
Source: BMC Geriatr. 2023 Dec 12;23:843. doi: 10.1186/s12877-023-04554-1 (PMC10717397; doi:10.1186/s12877-023-04554-1)
Supplement: Supplementary file 1 — Supplementary Material 1 [file 12877_2023_4554_MOESM1_ESM.docx]

**Supplemental Files**

*Frailty, cognitive impairment, and depressive symptoms in Chinese older adults: An eight-year multi-trajectory analysis*

|  | **Page** |
| --- | --- |
| *Main analysis (N=6,106)* |  |
| Supplemental Figure 1. Sample Flowchart (2011-2018; main analysis sample) | S-2 |
| Supplemental Table 1. Distribution of frailty index indicators (2011-2018; main analysis sample) | S-3, 4 |
| Supplemental Table 2. Distribution of frailty index, cognition measures, and CES-D scores (2011-2018; main analysis sample) | S-5 |
| Supplemental Table 3. Fit statistics of trajectory models of frailty, cognitive impairment, and depression (2011-2018; main analysis sample) | S-6 |
| Supplemental Table 4. Parameters of the best-fitting multi-trajectory model of frailty, cognitive impairment, and depressive symptoms (2011-2018; main analysis sample) | S-7 |
| Supplemental Table 5. Baseline characteristics by the trajectories of frailty, cognitive impairment, and depressive symptoms in Chinese older adults (2011-2018; main analysis sample) | S-8, 9 |
| Supplemental Table 6. Association between baseline characteristics and the trajectories of frailty, cognitive impairment, and depressive symptoms in older adults in China (2011-2018; main analysis sample): Original P-values and P-values corrected for multiple comparisons | S-10, 11 |
| *Sensitivity analysis (N=5,182)* |  |
| Supplemental Figure 2. Sample Flowchart (2011-2018; sensitivity analysis sample) | S-12 |
| Supplemental Figure 3. Trajectories of frailty, cognitive impairment, and depressive symptoms in Chinese older adults (2011-2018; sensitivity analysis sample) | S-13 |
| Supplemental Table 7. Baseline characteristics by the trajectories of frailty, cognitive impairment, and depressive symptoms in Chinese older adults (2011-2018; sensitivity analysis sample) | S-14, 15 |
| Supplemental Table 8. Distribution of frailty index indicators (2011-2018; sensitivity analysis sample) | S-16, 17 |
| Supplemental Table 9. Distribution of frailty index, cognition measures, and CES-D scores (2011-2018; sensitivity analysis sample) | S-18 |
| Supplemental Table 10. Fit statistics of trajectory models of frailty, cognitive impairment, and depression (2011-2018; sensitivity analysis sample) | S-19 |
| Supplemental Table 11. Parameters of the best-fitting multi-trajectory model of frailty, cognitive impairment, and depressive symptoms (2011-2018; sensitivity analysis sample) | S-20 |
| Supplemental Table 12. Association between baseline characteristics and the trajectories of frailty, cognitive impairment, and depressive symptoms in Chinese older adults (2011-2018; sensitivity analysis sample) | S-21, 22 |
| Supplemental Table 13. Association between baseline characteristics and the trajectories of frailty, cognitive impairment, and depressive symptoms in Chinese older adults (2011-2018; sensitivity analysis sample): Original P-values and P-values corrected for multiple comparisons | S-23, 24 |

**Supplemental Figure 1. Sample Flowchart (2011-2018; main analysis sample)**

CHARLS participants Wave 1 to 4 (2011-2018)

(n = 25,586)

Participated in Wave 1 and at least one other wave

(n = 16,448)

Aged 60 years or above in Wave 1

(n = 6,651)

Had non-missing values for frailty ^a^, cognition ^b^, and depressive symptoms ^c^ for at least 2 waves

(n = 6,106)

**Notes.**

^a^ frailty index (FI) was constructed with 30 indicators, including 13 on illness diagnoses from physicians, 5 on disabilities, and 12 on limitations with activities of daily living (ADLs) and instrumental activities of daily living (IADLs). Total score was divided by 30 and the final index ranges between 0 to 1.

^b^ Cognitive function was measured with episodic memory (range: 0-10), using the average score of immediate word recall (range: 0-10) and delayed word recall (range: 0-10), orientation and attention, using items from Telephone Interview of Cognitive Status (TICS-10) on naming of date, day of the week, and season (range: 0-5) and serial 7’s (range: 0-5), and visuoconstruction, using figure drawing (range: 0-1). Scores were summed to create an overall cognitive function score (range: 0-21)

^c^ Depressive symptoms were measured by the validated Chinese version of the 10-item Center for Epidemiologic Studies Depression Scale. Total score ranges between 0 to 30.

**Supplemental Table 1. Distribution of frailty index indicators (2011-2018; main analysis sample)**

|  |  |  | **Wave 1** | **Wave 2** | **Wave 3** | **Wave 4** |
| --- | --- | --- | --- | --- | --- | --- |
|  |  |  | (%) | (%) | (%) | (%) |
| Diagnosis ^a^ | Hypertension | | 33.3 | 37.1 | 43.9 | 51.6 |
|  | Diabetes or high blood sugar | | 7.5 | 9.3 | 11.9 | 15.5 |
|  | Cancer or malignant tumor | | 0.8 | 1.1 | 1.7 | 2.7 |
|  | Chronic lung disease | | 13.2 | 14.9 | 19.1 | 22.5 |
|  | Heart attack, coronary heart disease, angina, congestive heart failure or other heart problems | | 15.8 | 17.9 | 24.0 | 27.9 |
|  | Stroke | | 3.5 | 4.3 | 5.7 | 11.4 |
|  | Emotional, nervous, psychiatric problems | | 1.4 | 1.7 | 2.5 | 3.7 |
|  | Arthritis or rheumatism | | 37.1 | 39.2 | 50.0 | 47.8 |
|  | Dyslipidemia | | 10.5 | 13.9 | 19.9 | 23.5 |
|  | Liver disease | | 3.5 | 4.6 | 6.7 | 7.3 |
|  | Kidney disease | | 6.1 | 7.4 | 11.5 | 12.2 |
|  | Stomach or other digestive disease | | 21.7 | 24.1 | 32.6 | 32.8 |
|  | Asthma | | 6.2 | 6.9 | 9.1 | 9.9 |
| Disability ^b^ | Physical disabilities | | 4.4 | 7.2 | 10.7 | 13.3 |
|  | Brain damage/mental retardation | | 2.4 | 5.5 | 8.9 | 12.7 |
|  | Vision problem | | 8.5 | 14.2 | 20.6 | 24.6 |
|  | Hearing problem | | 12.5 | 20.6 | 29.2 | 34.9 |
|  | Speech impediment | | 0.2 | 0.5 | 1.2 | 1.9 |
| Functional limitation ^c^ | Doing household chores | |  |  |  |  |
|  |  | Have difficulty but can still do it | 6.5 | 6.7 | 8.8 | 9.8 |
|  |  | Have difficulty and need help | 1.6 | 2.1 | 3.2 | 5.2 |
|  |  | Cannot do it | 3.6 | 6.0 | 8.9 | 10.9 |
|  | Preparing hot meals | |  |  |  |  |
|  |  | Have difficulty but can still do it | 5.3 | 4.4 | 5.1 | 6.3 |
|  |  | Have difficulty and need help | 1.6 | 1.7 | 2.2 | 3.5 |
|  |  | Cannot do it | 4.4 | 6.9 | 8.9 | 11.2 |
|  | Managing assets | |  |  |  |  |
|  |  | Have difficulty but can still do it | 5.1 | 2.9 | 3.9 | 3.8 |
|  |  | Have difficulty and need help | 2.7 | 2.0 | 2.7 | 3.7 |
|  |  | Cannot do it | 7.0 | 8.0 | 8.3 | 10.7 |
|  | Taking medications | |  |  |  |  |
|  |  | Have difficulty but can still do it | 4.1 | 2.1 | 2.6 | 2.6 |
|  |  | Have difficulty and need help | 2.4 | 2.2 | 2.6 | 4.2 |
|  |  | Cannot do it | 1.0 | 1.2 | 1.6 | 2.4 |
|  | Shopping because of health and memory problems | |  |  |  |  |
|  |  | Have difficulty but can still do it | 4.6 | 3.0 | 3.2 | 3.9 |
|  |  | Have difficulty and need help | 1.9 | 1.8 | 2.1 | 3.1 |
|  |  | Cannot do it | 4.5 | 6.8 | 8.7 | 11.0 |
|  | Running or jogging About 1 kilometer | |  |  |  |  |
|  |  | Have difficulty but can still do it | 10.3 | 8.0 | 7.4 | 6.9 |
|  |  | Have difficulty and need help | 3.3 | 1.6 | 1.0 | 1.3 |
|  |  | Cannot do it | 49.8 | 56.3 | 59.7 | 64.3 |
|  | Getting up from a chair after sitting | |  |  |  |  |
|  |  | Have difficulty but can still do it | 28.7 | 30.7 | 32.0 | 36.4 |
|  |  | Have difficulty and need help | 2.8 | 2.2 | 3.2 | 3.5 |
|  |  | Cannot do it | 1.3 | 1.6 | 2.7 | 2.8 |
|  | Climbing several flights of stairs without rest | |  |  |  |  |
|  |  | Have difficulty but can still do it | 28.9 | 27.4 | 28.1 | 29.8 |
|  |  | Have difficulty and need help | 4.9 | 3.8 | 3.6 | 4.3 |
|  |  | Cannot do it | 15.1 | 19.7 | 21.7 | 25.8 |
|  | Stooping, kneeling, or crouching | |  |  |  |  |
|  |  | Have difficulty but can still do it | 23.5 | 24.2 | 24.0 | 26.4 |
|  |  | Have difficulty and need help | 3.8 | 3.1 | 2.8 | 3.3 |
|  |  | Cannot do it | 9.0 | 13.0 | 17.4 | 21.0 |
|  | Reaching or extending your arms above shoulder | |  |  |  |  |
|  |  | Have difficulty but can still do it | 6.3 | 5.3 | 6.0 | 6.1 |
|  |  | Have difficulty and need help | 1.3 | 0.7 | 0.8 | 1.1 |
|  |  | Cannot do it | 4.9 | 7.7 | 9.6 | 12.6 |
|  | Lifting or carrying weights over 10 jin (about 5 kilograms) | |  |  |  |  |
|  |  | Have difficulty but can still do it | 6.3 | 6.0 | 6.6 | 8.0 |
|  |  | Have difficulty and need help | 1.4 | 1.4 | 1.6 | 2.5 |
|  |  | Cannot do it | 7.7 | 12.3 | 15.6 | 19.5 |
|  | Picking up a small coin from a table | |  |  |  |  |
|  |  | Have difficulty but can still do it | 2.6 | 2.5 | 3.5 | 3.6 |
|  |  | Have difficulty and need help | 0.4 | 0.3 | 0.5 | 0.9 |
|  |  | Cannot do it | 1.6 | 2.7 | 3.8 | 5.0 |

**Notes.**

^a^ Self-reported diagnoses, based on respondents’ answers to the question: “Have you been diagnosed with [conditions] by a doctor.”

^b^ Self-reported disabilities, based on respondents’ answers to the question: “Do you have the following disabilities?”

^c^ Self-reported functional limitations, based on respondents’ answers to the questions “Do you have difficulty with…?”

**Supplemental Table 2. Distribution of frailty index, cognition measures, and CES-D scores (2011-2018; main analysis sample)**

|  |  | **Wave 1** | | **Wave 2** | | **Wave 3** | | **Wave 4** | |
| --- | --- | --- | --- | --- | --- | --- | --- | --- | --- |
| Measures (total score range) | | Mean | (SD) | Mean | (SD) | Mean | (SD) | Mean | (SD) |
| Frailty index (0-1) ^a^ | | 0.12 | (0.10) | 0.14 | (0.11) | 0.18 | (0.12) | 0.21 | (0.14) |
| Cognition (0-21) ^b^ | | 9.79 | (4.44) | 9.63 | (4.62) | 9.10 | (4.66) | 7.96 | (5.05) |
|  | Average of immediate and delayed word recall (0-10) | 3.25 | (1.60) | 3.13 | (1.68) | 2.59 | (1.78) | 2.74 | (2.06) |
|  | Numerical ability (0-5) | 3.38 | (1.71) | 3.40 | (1.66) | 3.43 | (1.61) | 3.30 | (1.64) |
|  | Orientation (0-5) | 3.79 | (1.28) | 3.86 | (1.29) | 3.71 | (1.35) | 3.23 | (1.49) |
|  | Figure drawing (0-1) | 0.55 | (0.50) | 0.54 | (0.50) | 0.50 | (0.50) | 0.50 | (0.50) |
| CES-D score (0-30) ^c^ | | 8.94 | (6.41) | 8.12 | (5.83) | 8.62 | (6.61) | 8.93 | (6.62) |

**Notes.** CES-D = Center for Epidemiological Studies Depression.

^a^ Higher score indicates greater frailty.

^b^ Higher scores indicate better cognitive function.

^c^ Higher scores indicate more frequent depressive symptoms.

**Supplemental Table 3. Fit statistics of trajectory models of frailty, cognitive impairment, and depression (2011-2018; main analysis sample)**

| # Trajectory groups ^a^ | BIC | Sample size BIC | AIC | Group membership % | Group AvePP ^b^ | OCC ^c^ |
| --- | --- | --- | --- | --- | --- | --- |
| 2 | -109068.56 | -109035.53 | -108941.49 | 64.28 (a) | 0.965 (b) | 15.10 |
|  |  |  |  | 35.72 | 0.944 | 30.35 |
| 3 | -105757.06 | -105708.70 | -105571.00 | 29.85 | 0.915 | 25.14 |
|  |  |  |  | 47.78 | 0.957 | 24.32 |
|  |  |  |  | 22.37 | 0.927 | 43.81 |
| 4 | -103656.21 | -103592.51 | -103411.15 | 29.35 | 0.927 | 30.53 |
|  |  |  |  | 36.62 | 0.938 | 26.22 |
|  |  |  |  | 19.97 | 0.895 | 34.27 |
|  |  |  |  | 14.06 | 0.939 | 93.40 |
| 5 | -102395.12 | -102316.08 | -102091.06 | 22.88 | 0.899 | 29.88 |
|  |  |  |  | 16.37 | 0.900 | 45.74 |
|  |  |  |  | 32.74 | 0.938 | 30.93 |
|  |  |  |  | 20.80 | 0.898 | 33.65 |
|  |  |  |  | 7.21 | 0.932 | 176.29 |
| 6 | -101824.84 | -101730.47 | -101461.79 | 20.87 | 0.894 | 32.10 |
|  |  |  |  | 17.40 | 0.897 | 41.27 |
|  |  |  |  | 26.97 | 0.914 | 28.81 |
|  |  |  |  | 21.60 | 0.856 | 21.51 |
|  |  |  |  | 5.68 | 0.928 | 212.92 |
|  |  |  |  | 7.48 | 0.870 | 82.41 |

**Notes.** BIC = Bayesian information criterion; AIC = Akaike information criterion; AvePP = Average posterior probability; OCC = Odds of correct classification.

^a^ For all models, the starting shape parameter for all trajectory groups was set to cubic.

^b^ Group AvePP of assignment: Based on the maximum probability assignment rule, individuals will be assigned to a group according to the largest posterior probability. For all the individuals assigned to a certain group, an AvePP will be calculated. For each trajectory group, an AvePP of assignment > 0.7 is indicative of good certainty of group assignments.

^c^ OCC: The numerator is the odds of a correct classification into a certain group based on the model, and the denominator is the correct classification into that group based on random assignment, essentially, OCC = [b/(1-b)]/[a/(1-a)]. For each trajectory group, OCC >= 5 suggests high assignment accuracy.

**Supplemental Table 4. Parameters of the best-fitting multi-trajectory model of frailty, cognitive impairment, and depressive symptoms (2011-2018; main analysis sample)**

|  | Parameter | Estimate | SE | t | P-value |
| --- | --- | --- | --- | --- | --- |
| Frailty | | | | | |
| Trajectory 1 | Intercept | 0.261 | 0.009 | 28.270 | 0.000 |
|  | Linear | -0.022 | 0.010 | -2.191 | 0.029 |
|  | Quadratic | 0.015 | 0.003 | 5.551 | 0.000 |
|  | Cubic | -0.001 | 0.000 | -5.994 | 0.000 |
| Trajectory 2 | Intercept | 0.169 | 0.008 | 21.889 | 0.000 |
|  | Linear | -0.006 | 0.008 | -0.691 | 0.490 |
|  | Quadratic | 0.007 | 0.002 | 2.975 | 0.003 |
|  | Cubic | -0.001 | 0.000 | -2.970 | 0.003 |
| Trajectory 3 | Intercept | 0.098 | 0.006 | 15.492 | 0.000 |
|  | Linear | -0.010 | 0.007 | -1.500 | 0.134 |
|  | Quadratic | 0.006 | 0.002 | 3.071 | 0.002 |
|  | Cubic | 0.000 | 0.000 | -2.799 | 0.005 |
| Trajectory 4 | Intercept | 0.054 | 0.006 | 9.610 | 0.000 |
|  | Linear | -0.005 | 0.006 | -0.886 | 0.376 |
|  | Quadratic | 0.004 | 0.002 | 2.337 | 0.019 |
|  | Cubic | 0.000 | 0.000 | -2.326 | 0.020 |
| Cognitive impairment | | | | | |
| Trajectory 1 | Intercept | 6.423 | 0.130 | 49.491 | 0.000 |
|  | Linear | -0.397 | 0.027 | -14.929 | 0.000 |
| Trajectory 2 | Intercept | 11.120 | 0.309 | 35.936 | 0.000 |
|  | Linear | 0.979 | 0.335 | 2.926 | 0.003 |
|  | Quadratic | -0.272 | 0.092 | -2.966 | 0.003 |
|  | Cubic | 0.017 | 0.007 | 2.432 | 0.015 |
| Trajectory 3 | Intercept | 6.335 | 0.133 | 47.534 | 0.000 |
|  | Linear | -0.055 | 0.070 | -0.780 | 0.435 |
|  | Quadratic | -0.044 | 0.008 | -5.835 | 0.000 |
| Trajectory 4 | Intercept | 12.913 | 0.116 | 111.260 | 0.000 |
|  | Linear | 0.104 | 0.061 | 1.713 | 0.087 |
|  | Quadratic | -0.035 | 0.007 | -5.233 | 0.000 |
| Depressive symptoms | | | | | |
| Trajectory 1 | Intercept | 18.174 | 0.697 | 26.061 | 0.000 |
|  | Linear | -5.195 | 0.760 | -6.833 | 0.000 |
|  | Quadratic | 1.424 | 0.210 | 6.793 | 0.000 |
|  | Cubic | -0.104 | 0.016 | -6.534 | 0.000 |
| Trajectory 2 | Intercept | 12.968 | 0.589 | 22.008 | 0.000 |
|  | Linear | -2.573 | 0.631 | -4.078 | 0.000 |
|  | Quadratic | 0.659 | 0.173 | 3.813 | 0.000 |
|  | Cubic | -0.045 | 0.013 | -3.433 | 0.001 |
| Trajectory 3 | Intercept | 10.758 | 0.481 | 22.360 | 0.000 |
|  | Linear | -2.289 | 0.522 | -4.386 | 0.000 |
|  | Quadratic | 0.538 | 0.143 | 3.769 | 0.000 |
|  | Cubic | -0.035 | 0.011 | -3.258 | 0.001 |
| Trajectory 4 | Intercept | 5.474 | 0.219 | 24.968 | 0.000 |
|  | Linear | -0.249 | 0.117 | -2.133 | 0.033 |
|  | Quadratic | 0.030 | 0.013 | 2.411 | 0.016 |

**Notes.** Trajectory 1 = Worsening frailty, worsening cognitive impairment, depression; Trajectory 2 = Declining pre-frailty, declining cognition, borderline depression; Trajectory 3 = Pre-frailty, worsening cognitive impairment, no depression; Trajectory 4 = Physically robust, declining cognition, no depression.

**Supplemental Table 5. Baseline characteristics by the trajectories of frailty, cognitive impairment, and depressive symptoms in Chinese older adults (2011-2018; main analysis sample)**

|  |  | **All** | **By trajectories of frailty, cognitive impairment, and depressive symptoms** | | | |
| --- | --- | --- | --- | --- | --- | --- |
|  |  |  | Worsening frailty, worsening cognitive impairment, depression | Declining pre-frailty, declining cognition, borderline depression | Pre-frailty,  worsening cognitive impairment, no depression | Physically robust, declining cognition, no depression |
|  |  | (n=6,106) | (n=853) | (n=1,193) | (n=1,796) | (n=2,264) |
| **Age** (median, IQR) | | 66.0 (9.0) | 70.0 (10.0) | 65.0 (7.0) | 68.0 (10.0) | 64.0 (7.0) |
| **Female** (%) | | 49.7 | 67.9 | 46.1 | 66.2 | 31.8 |
| ***Human capital*** | |  |  |  |  |  |
| **Education** (%) | |  |  |  |  |  |
|  | No formal education | 35.2 | 64.0 | 13.4 | 68.2 | 9.5 |
|  | Less than elementary school | 20.8 | 17.8 | 26.2 | 20.1 | 19.7 |
|  | Elementary school or higher | 44.0 | 18.2 | 60.4 | 11.8 | 70.8 |
| **Work status** (%) | |  |  |  |  |  |
|  | Currently working | 49.7 | 30.1 | 44.7 | 56.2 | 54.3 |
|  | Retired | 47.9 | 66.4 | 51.8 | 41.5 | 44.0 |
|  | Not employed/Never worked | 2.5 | 3.5 | 3.5 | 2.3 | 1.7 |
| ***Social capital*** | |  |  |  |  |  |
| Not married ^a^ (%) | | 19.8 | 30.1 | 16.8 | 26.5 | 12.2 |
| Empty nester ^b^ (%) | | 54.0 | 52.9 | 55.8 | 52.0 | 54.9 |
| No participation in any social activities (%) | | 54.8 | 64.1 | 53.5 | 61.2 | 47.0 |
| ***Financial capital*** | |  |  |  |  |  |
| **Childhood family financial situation ^c^** (%) | |  |  |  |  |  |
|  | A lot/somewhat better off | 9.0 | 7.0 | 9.5 | 7.9 | 10.3 |
|  | Same | 50.1 | 41.8 | 48.5 | 48.8 | 55.2 |
|  | Somewhat/A lot worse off | 40.9 | 51.3 | 41.9 | 43.3 | 34.6 |
| **Per capita household consumption at baseline** (in Chinese Yuan; %) | |  |  |  |  |  |
|  | Quartile 1 [ 0.0 - 2638.0) | 25.0 | 29.3 | 19.2 | 34.0 | 19.5 |
|  | Quartile 2 [2638.0 - 4458.8) | 25.0 | 27.3 | 22.9 | 29.3 | 22.0 |
|  | Quartile 3 [4458.8 - 8149.0) | 25.0 | 23.9 | 28.4 | 22.5 | 25.5 |
|  | Quartile 4 [8149.0 - 222,640] | 25.0 | 19.6 | 29.5 | 14.2 | 33.0 |
| ***Health capital*** | |  |  |  |  |  |
| **Childhood health ^d^** (%) | |  |  |  |  |  |
|  | Much/somewhat healthier | 34.6 | 29.3 | 31.7 | 32.0 | 40.1 |
|  | About average | 52.8 | 53.0 | 52.5 | 54.9 | 51.2 |
|  | Somewhat less healthy/Much less healthy | 12.6 | 17.7 | 15.8 | 13.1 | 8.7 |
| **Self-rated health at baseline** (%) | |  |  |  |  |  |
|  | Very good/good | 19.4 | 5.8 | 7.4 | 19.8 | 30.5 |
|  | Fair | 49.3 | 27.4 | 45.0 | 52.5 | 57.2 |
|  | Poor/very poor | 31.4 | 66.8 | 47.6 | 27.7 | 12.3 |

| ***Structural factor*** |  |
| --- | --- |

| ***Hukou* and residence** (%) | |  |  |  |  |  |
| --- | --- | --- | --- | --- | --- | --- |
|  | Rural *hukou*, rural residence | 59.2 | 71.5 | 54.0 | 72.9 | 46.4 |
|  | Rural *hukou*, urban residence | 17.5 | 16.7 | 14.7 | 19.1 | 18.1 |
|  | Urban *hukou*, rural residence | 2.9 | 1.3 | 3.8 | 1.2 | 4.3 |
|  | Urban *hukou*, urban residence | 20.4 | 10.6 | 27.5 | 6.8 | 31.2 |

**Notes.** IQR = interquartile range.

^a^ Including widowed, divorced, separated, and never married.

^b^ P = 0.1407, P-value for all other characteristics <.0001.

^c^ Self-rated family financial situation before age 17 years, compared to the average family in the same community/village. ^d^ Self-rated health before age 16 years, compared to other children of the same age.

**Supplemental Table 6. Association between baseline characteristics and the trajectories of frailty, cognitive impairment, and depressive symptoms in older adults in China (2011-2018; main analysis sample) ^a^: Original P-values and P-values corrected for multiple comparisons**

|  | | **Trajectories of frailty, cognitive impairment, and depressive symptoms** | | | | | |
| --- | --- | --- | --- | --- | --- | --- | --- |
|  | | (*Ref: Physically robust, declining cognition, no depression*) | | | | | |
|  | | Worsening frailty, worsening cognitive impairment, depression | | Declining pre-frailty, declining cognition, borderline depression | | Pre-frailty,  worsening cognitive impairment, no depression | |
|  | | Original ^b^ | Corrected ^c^ | Original ^b^ | Corrected ^c^ | Original ^b^ | Corrected ^c^ |
| **Age** (every 5-year increase; continuous) | | **0.000** | **0.000** | **0.000** | **0.000** | **0.000** | **0.000** |
| **Female** (ref: Male) | | **0.000** | **0.000** | **0.000** | **0.000** | **0.000** | **0.000** |
| ***Human capital*** | |  |  |  |  |  |  |
| **Education** (ref: Elementary school or higher) | |  |  |  |  |  |  |
|  | No formal education | **0.000** | **0.000** | **0.016** | 0.160 | **0.000** | **0.000** |
|  | Less than elementary school | **0.000** | **0.000** | **0.001** | **0.016** | **0.000** | **0.000** |
| **Work status** (ref: Currently working) | |  |  |  |  |  |  |
|  | Retired | **0.000** | **0.000** | **0.000** | **0.000** | 0.259 | 0.960 |
|  | Not employed/Never worked | **0.001** | **0.010** | **0.003** | **0.045** | 0.629 | 0.960 |
| ***Social capital*** | |  |  |  |  |  |  |
| **Not married ^a^** (ref: Married) | | **0.013** | 0.117 | 0.081 | 0.666 | **0.013** | 0.117 |
| **Empty nester** (ref: Living with children) | | 0.216 | 1.000 | 0.886 | 1.000 | 0.192 | 0.960 |
| **No participation in any social activities** (ref: Any social activity participation) | | **0.000** | **0.000** | **0.004** | 0.056 | **0.000** | **0.000** |
| ***Financial capital*** | |  |  |  |  |  |  |
| **Childhood family financial situation ^b^** (ref: Same) | |  |  |  |  |  |  |
|  | A lot better off/somewhat better off | 0.243 | 1.000 | 0.155 | 1.000 | 0.203 | 0.960 |
|  | Somewhat worse off/A lot worse off | **0.000** | **0.000** | **0.005** | 0.056 | 0.127 | 0.762 |
| **Per capita household consumption** (ref: Quartile 4) | |  |  |  |  |  |  |
|  | Quartile 1 | 0.161 | 1.000 | 0.817 | 1.000 | **0.002** | **0.022** |
|  | Quartile 2 | 0.243 | 0.930 | 0.628 | 1.000 | **0.000** | **0.000** |
|  | Quartile 3 | 0.075 | 1.000 | 0.181 | 1.000 | 0.086 | 0.602 |
| ***Health capital*** | |  |  |  |  |  |  |
| **Childhood health ^c^** (ref: About average) | |  |  |  |  |  |  |
|  | Much healthier/somewhat healthier | **0.016** | 0.128 | 0.074 | 0.666 | **0.002** | **0.022** |
|  | Somewhat less healthy/Much less healthy | 0.077 | 0.539 | **0.004** | 0.056 | 0.442 | 0.960 |
| **Self-rated health** (ref: Fair) | |  |  |  |  |  |  |
|  | Very good/good | **0.000** | **0.000** | **0.000** | **0.000** | **0.000** | **0.000** |
|  | Poor/very poor | **0.000** | **0.000** | **0.000** | **0.000** | **0.000** | **0.000** |
| ***Structural factor*** | |  |  |  |  |  |  |
| ***Hukou* and residence** (ref: Urban *hukou*, urban residence) | |  |  |  |  |  |  |
|  | Rural *hukou*, rural residence | **0.000** | **0.000** | **0.004** | 0.056 | **0.000** | **0.000** |
|  | Rural *hukou*, urban residence | **0.000** | **0.000** | 0.525 | 1.000 | **0.000** | **0.000** |
|  | Urban *hukou*, rural residence | 0.233 | 1.000 | 0.581 | 1.000 | 0.063 | 0.504 |

**Notes.**

^a^ Associations in adjusted odds ratios and 95% confidence intervals were summarized in Table 2 in the main manuscript.

^b^ Original P-values

^c^ P-values corrected by the Holm step-down method for multiple comparisons.

**Supplemental Figure 2. Sample Flowchart (2011-2018; sensitivity analysis sample)**

CHARLS participants Wave 1 to 4 (2011-2018)

(n = 25,586)

Participated in Wave 1 and at least one other wave

(n = 16,448)

Aged 60 years or above in Wave 1

(n = 6,651)

Had non-missing values for frailty ^a^, cognition ^b^, and depressive symptoms ^c^ for at least 3 waves

(n = 5,182)

**Notes.**

^a^ frailty index (FI) was constructed with 30 indicators, including 13 on illness diagnoses from physicians, 5 on disabilities, and 12 on limitations with activities of daily living (ADLs) and instrumental activities of daily living (IADLs). Total score was divided by 30 and the final index ranges between 0 to 1.

^b^ Cognitive function was measured with episodic memory (range: 0-10), using the average score of immediate word recall (range: 0-10) and delayed word recall (range: 0-10), orientation and attention, using items from Telephone Interview of Cognitive Status (TICS-10) on naming of date, day of the week, and season (range: 0-5) and serial 7’s (range: 0-5), and visuoconstruction, using figure drawing (range: 0-1). Scores were summed to create an overall cognitive function score (range: 0-21)

^c^ Depressive symptoms were measured by the validated Chinese version of the 10-item Center for Epidemiologic Studies Depression Scale. Total score ranges between 0 to 30.

**Supplemental Figure 3. Trajectories of frailty, cognitive impairment, and depressive symptoms in Chinese older adults (2011-2018; sensitivity analysis sample)**

**
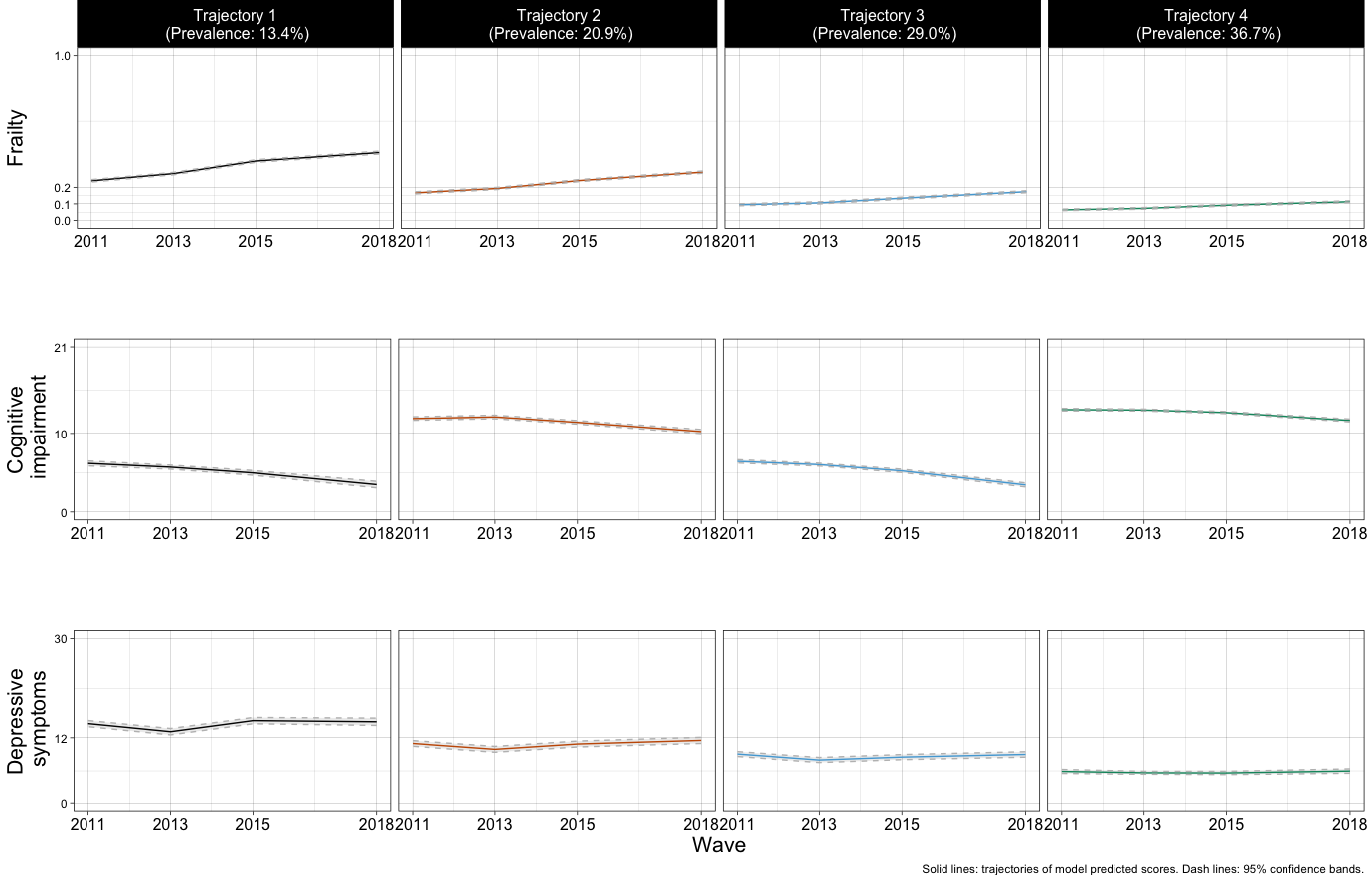
**

**Notes.** Trajectory 1 = Worsening frailty, worsening cognitive impairment, depression; Trajectory 2 = Declining pre-frailty, declining cognition, borderline depression; Trajectory 3 = Pre-frailty, worsening cognitive impairment, no depression; Trajectory 4 = Physically robust, declining cognition, no depression.

**Supplemental Table 7. Baseline characteristics by the trajectories of frailty, cognitive impairment, and depressive symptoms in Chinese older adults (2011-2018; sensitivity analysis sample)**

|  |  | **All** | **By trajectories of frailty, cognitive impairment, and depressive symptoms** | | | |
| --- | --- | --- | --- | --- | --- | --- |
|  |  |  | Worsening frailty, worsening cognitive impairment, depression | Declining pre-frailty, declining cognition, borderline depression | Pre-frailty,  worsening cognitive impairment, no depression | Physically robust, declining cognition, no depression |
|  |  | (n=5,182) | (n=696) | (n=1,057) | (n=1,503) | (n=1,926) |
| **Age** (median, IQR) | | 66.0 (9.0) | 69.0 (9.0) | 65.0 (7.0) | 67.0 (9.0) | 64.0 (6.0) |
| **Female** (%) | | 49.8 | 70.3 | 45.9 | 66.5 | 31.6 |
| ***Human capital*** | |  |  |  |  |  |
| **Education** (%) | |  |  |  |  |  |
|  | No formal education | 34.0 | 63.9 | 12.3 | 67.8 | 8.8 |
|  | Less than elementary school | 21.2 | 18.8 | 27.1 | 20.2 | 19.6 |
|  | Elementary school or higher | 44.8 | 17.2 | 60.6 | 12.0 | 71.6 |
| **Work status** (%) | |  |  |  |  |  |
|  | Currently working | 52.8 | 36.2 | 47.5 | 59.8 | 56.2 |
|  | Retired | 45.0 | 60.7 | 49.8 | 38.3 | 41.9 |
|  | Not employed/Never worked | 2.3 | 3.1 | 2.7 | 2.0 | 1.9 |
| ***Social capital*** | |  |  |  |  |  |
| Not married ^a^ (%) | | 18.8 | 27.4 | 16.6 | 25.0 | 11.9 |
| Empty nester ^b^ (%) | | 53.9 | 52.9 | 55.5 | 51.8 | 54.9 |
| No participation in any social activities (%) | | 45.5 | 64.2 | 52.5 | 60.9 | 47.1 |
| ***Financial capital*** | |  |  |  |  |  |
| **Childhood family financial situation ^c^** (%) | |  |  |  |  |  |
|  | A lot/somewhat better off | 8.3 | 4.9 | 8.9 | 7.6 | 9.7 |
|  | Same | 50.9 | 43.7 | 49.3 | 49.1 | 55.8 |
|  | Somewhat/A lot worse off | 40.8 | 51.4 | 41.9 | 43.3 | 34.5 |
| **Per capita household consumption at baseline** (in Chinese Yuan; %) | |  |  |  |  |  |
|  | Quartile 1 [ 0.0 - 2638.0) | 25.0 | 29.2 | 18.9 | 34.2 | 19.9 |
|  | Quartile 2 [2638.0 - 4458.8) | 25.0 | 26.8 | 24.3 | 28.6 | 22.1 |
|  | Quartile 3 [4458.8 - 8149.0) | 25.0 | 24.1 | 27.7 | 23.0 | 25.4 |
|  | Quartile 4 [8149.0 - 222,640] | 25.0 | 19.9 | 29.1 | 14.2 | 32.7 |
| ***Health capital*** | |  |  |  |  |  |
| **Childhood health ^d^** (%) | |  |  |  |  |  |
|  | Much/somewhat healthier | 34.7 | 28.9 | 31.7 | 32.8 | 39.8 |
|  | About average | 52.8 | 52.2 | 53.0 | 54.4 | 51.7 |
|  | Somewhat less healthy/Much less healthy | 12.6 | 18.9 | 15.3 | 12.9 | 8.5 |
| **Self-rated health at baseline** (%) | |  |  |  |  |  |
|  | Very good/good | 19.5 | 4.9 | 7.3 | 20.6 | 30.6 |
|  | Fair | 49.6 | 27.4 | 45.6 | 52.2 | 57.6 |
|  | Poor/very poor | 30.9 | 67.7 | 47.1 | 27.2 | 11.8 |
| ***Structural factor*** | |  |  |  |  |  |
| ***Hukou* and residence** (%) | |  |  |  |  |  |
|  | Rural *hukou*, rural residence | 60.6 | 72.3 | 55.9 | 74.4 | 48.3 |
|  | Rural *hukou*, urban residence | 17.2 | 17.0 | 14.9 | 18.4 | 17.7 |
|  | Urban *hukou*, rural residence | 3.0 | 1.4 | 3.9 | 1.3 | 4.5 |
|  | Urban *hukou*, urban residence | 19.1 | 9.3 | 25.4 | 5.9 | 29.5 |

**Notes.** IQR = interquartile range.

^a^ Including widowed, divorced, separated, and never married.

^b^ P = 0.1853, P-value for all other characteristics <.0001.

^c^ Self-rated family financial situation before age 17 years, compared to the average family in the same community/village. ^d^ Self-rated health before age 16 years, compared to other children of the same age.

**Supplemental Table 8. Distribution of frailty index indicators (2011-2018; sensitivity analysis sample)**

|  |  |  | **Wave 1** | **Wave 2** | **Wave 3** | **Wave 4** |
| --- | --- | --- | --- | --- | --- | --- |
|  |  |  | (%) | (%) | (%) | (%) |
| Diagnosis ^a^ | Hypertension | | 32.6 | 37.1 | 43.9 | 51.6 |
|  | Diabetes or high blood sugar | | 7.4 | 9.3 | 11.9 | 15.5 |
|  | Cancer or malignant tumor | | 0.8 | 1.1 | 1.7 | 2.7 |
|  | Chronic lung disease | | 12.7 | 14.9 | 19.1 | 22.5 |
|  | Heart attack, coronary heart disease, angina, congestive heart failure or other heart problems | | 15.4 | 17.9 | 24.0 | 27.9 |
|  | Stroke | | 3.2 | 4.3 | 5.7 | 11.4 |
|  | Emotional, nervous, psychiatric problems | | 1.4 | 1.7 | 2.5 | 3.7 |
|  | Arthritis or rheumatism | | 37.5 | 39.2 | 50.0 | 47.8 |
|  | Dyslipidemia | | 10.4 | 13.9 | 19.9 | 23.5 |
|  | Liver disease | | 3.4 | 4.6 | 6.7 | 7.3 |
|  | Kidney disease | | 6.1 | 7.4 | 11.5 | 12.2 |
|  | Stomach or other digestive disease | | 22.4 | 24.1 | 32.6 | 32.8 |
|  | Asthma | | 6.4 | 6.9 | 9.1 | 9.9 |
| Disability ^b^ | Physical disabilities | | 4.1 | 7.2 | 10.7 | 13.3 |
|  | Brain damage/mental retardation | | 2.5 | 5.5 | 8.9 | 12.7 |
|  | Vision problem | | 8.0 | 14.2 | 20.6 | 24.6 |
|  | Hearing problem | | 11.5 | 20.6 | 29.2 | 34.9 |
|  | Speech impediment | | 0.2 | 0.5 | 1.2 | 1.9 |
| Functional limitation ^c^ | Doing household chores | |  |  |  |  |
|  |  | Have difficulty but can still do it | 6.5 | 6.6 | 8.7 | 10.0 |
|  |  | Have difficulty and need help | 1.5 | 1.9 | 3.2 | 5.0 |
|  |  | Cannot do it | 2.8 | 4.5 | 7.4 | 9.9 |
|  | Preparing hot meals | |  |  |  |  |
|  |  | Have difficulty but can still do it | 5.2 | 4.3 | 5.0 | 6.3 |
|  |  | Have difficulty and need help | 1.5 | 1.5 | 2.0 | 3.4 |
|  |  | Cannot do it | 3.4 | 5.4 | 7.4 | 10.2 |
|  | Managing assets | |  |  |  |  |
|  |  | Have difficulty but can still do it | 5.0 | 2.8 | 3.8 | 3.9 |
|  |  | Have difficulty and need help | 2.7 | 1.9 | 2.5 | 3.6 |
|  |  | Cannot do it | 6.3 | 6.9 | 7.1 | 9.8 |
|  | Taking medications | |  |  |  |  |
|  |  | Have difficulty but can still do it | 4.1 | 2.2 | 2.5 | 2.6 |
|  |  | Have difficulty and need help | 2.4 | 1.9 | 2.1 | 3.9 |
|  |  | Cannot do it | 0.8 | 0.8 | 1.1 | 1.9 |
|  | Shopping because of health and memory problems | |  |  |  |  |
|  |  | Have difficulty but can still do it | 4.8 | 3.0 | 3.2 | 4.0 |
|  |  | Have difficulty and need help | 1.8 | 1.7 | 2.0 | 2.9 |
|  |  | Cannot do it | 3.5 | 5.2 | 7.1 | 10.0 |
|  | Running or jogging About 1 kilometer | |  |  |  |  |
|  |  | Have difficulty but can still do it | 10.3 | 8.3 | 7.6 | 7.0 |
|  |  | Have difficulty and need help | 3.3 | 1.4 | 1.0 | 1.3 |
|  |  | Cannot do it | 47.9 | 54.5 | 58.5 | 63.6 |
|  | Getting up from a chair after sitting | |  |  |  |  |
|  |  | Have difficulty but can still do it | 28.7 | 30.9 | 32.0 | 36.6 |
|  |  | Have difficulty and need help | 2.5 | 1.8 | 2.9 | 3.4 |
|  |  | Cannot do it | 1.0 | 1.3 | 2.3 | 2.4 |
|  | Climbing several flights of stairs without rest | |  |  |  |  |
|  |  | Have difficulty but can still do it | 28.9 | 28.1 | 28.3 | 30.0 |
|  |  | Have difficulty and need help | 4.9 | 3.4 | 3.4 | 4.2 |
|  |  | Cannot do it | 13.8 | 18.0 | 20.3 | 25.1 |
|  | Stooping, kneeling, or crouching | |  |  |  |  |
|  |  | Have difficulty but can still do it | 23.8 | 24.3 | 24.4 | 26.4 |
|  |  | Have difficulty and need help | 3.7 | 2.8 | 2.6 | 3.2 |
|  |  | Cannot do it | 8.2 | 12.1 | 16.4 | 20.6 |
|  | Reaching or extending your arms above shoulder | |  |  |  |  |
|  |  | Have difficulty but can still do it | 6.2 | 5.2 | 6.1 | 6.1 |
|  |  | Have difficulty and need help | 1.3 | 0.7 | 0.8 | 1.0 |
|  |  | Cannot do it | 4.5 | 7.0 | 8.7 | 12.1 |
|  | Lifting or carrying weights over 10 jin (about 5 kilograms) | |  |  |  |  |
|  |  | Have difficulty but can still do it | 5.9 | 5.7 | 6.4 | 8.2 |
|  |  | Have difficulty and need help | 1.3 | 1.3 | 1.4 | 2.2 |
|  |  | Cannot do it | 6.7 | 10.9 | 14.1 | 18.5 |
|  | Picking up a small coin from a table | |  |  |  |  |
|  |  | Have difficulty but can still do it | 2.4 | 2.4 | 3.5 | 3.6 |
|  |  | Have difficulty and need help | 0.4 | 0.4 | 0.4 | 0.8 |
|  |  | Cannot do it | 1.4 | 2.2 | 3.4 | 4.6 |

**Notes.**

^a^ Self-reported diagnoses, based on respondents’ answers to the question: “Have you been diagnosed with [conditions] by a doctor.”

^b^ Self-reported disabilities, based on respondents’ answers to the question: “Do you have the following disabilities?”

^c^ Self-reported functional limitations, based on respondents’ answers to the questions “Do you have difficulty with…?”

**Supplemental Table 9. Distribution of frailty index, cognition measures, and CES-D scores (2011-2018; sensitivity analysis sample)**

|  |  | **Wave 1** | | **Wave 2** | | **Wave 3** | | **Wave 4** | |
| --- | --- | --- | --- | --- | --- | --- | --- | --- | --- |
| Measures (total score range) | | Mean | (SD) | Mean | (SD) | Mean | (SD) | Mean | (SD) |
| Frailty index (0-1) ^a^ | | 0.12 | (0.09) | 0.13 | (0.10) | 0.17 | (0.12) | 0.20 | (0.14) |
| Cognition (0-21) ^b^ | | 9.95 | (4.37) | 9.82 | (4.52) | 9.23 | (4.59) | 8.01 | (5.04) |
|  | Average of immediate and delayed word recall (0-10) | 3.27 | (1.59) | 3.18 | (1.66) | 2.62 | (1.77) | 2.74 | (2.06) |
|  | Numerical ability (0-5) | 3.42 | (1.69) | 3.43 | (1.64) | 3.44 | (1.60) | 3.30 | (1.64) |
|  | Orientation to date and time (0-5) | 3.81 | (1.26) | 3.89 | (1.26) | 3.73 | (1.34) | 3.23 | (1.48) |
|  | Figure drawing (0-1) | 0.56 | (0.50) | 0.56 | (0.50) | 0.51 | (0.50) | 0.50 | (0.50) |
| CES-D score (0-30) ^c^ | | 8.92 | (6.42) | 8.10 | (5.81) | 8.67 | (6.63) | 8.95 | (6.62) |

**Notes.** CES-D = Center for Epidemiological Studies Depression.

^a^ Higher score indicates greater frailty.

^b^ Higher scores indicate better cognitive function.

^c^ Higher scores indicate more frequent depressive symptoms.

**Supplemental Table 10. Fit statistics of trajectory models of frailty, cognitive impairment, and depression (2011-2018; sensitivity analysis sample)**

| # Trajectory groups ^a^ | | BIC | | Sample size BIC | | AIC | | Group membership % | | Group AvePP ^b^ | | OCC ^c^ |
| --- | --- | --- | --- | --- | --- | --- | --- | --- | --- | --- | --- | --- |
| 2 | -98919.89 | | -98885.99 | | -98794.25 | | 64.53 (a) | | 0.967 (b) | | 16.24 | |
|  |  | |  | |  | | 35.47 | | 0.945 | | 31.25 | |
| 3 | -95828.45 | | -95778.81 | | -95644.47 | | 29.50 | | 0.927 | | 30.32 | |
|  |  | |  | |  | | 48.05 | | 0.960 | | 25.74 | |
|  |  | |  | |  | | 22.44 | | 0.928 | | 44.65 | |
| 4 | -93877.63 | | -93812.25 | | -93635.32 | | 29.03 | | 0.934 | | 34.84 | |
|  |  | |  | |  | | 36.67 | | 0.942 | | 27.94 | |
|  |  | |  | |  | | 20.87 | | 0.908 | | 37.55 | |
|  |  | |  | |  | | 13.43 | | 0.938 | | 96.75 | |
| 5 | -92733.27 | | -92652.15 | | -92432.62 | | 23.12 | | 0.908 | | 32.87 | |
|  |  | |  | |  | | 15.63 | | 0.909 | | 54.07 | |
|  |  | |  | |  | | 33.02 | | 0.944 | | 34.04 | |
|  |  | |  | |  | | 21.79 | | 0.907 | | 35.21 | |
|  |  | |  | |  | | 6.44 | | 0.944 | | 242.96 | |
| 6 | -92208.14 | | -92111.28 | | -91849.16 | | 20.37 | | 0.902 | | 36.08 | |
|  |  | |  | |  | | 17.06 | | 0.910 | | 48.95 | |
|  |  | |  | |  | | 24.95 | | 0.918 | | 33.87 | |
|  |  | |  | |  | | 22.76 | | 0.852 | | 19.49 | |
|  |  | |  | |  | | 5.83 | | 0.930 | | 213.06 | |
|  |  | |  | |  | | 9.03 | | 0.881 | | 74.44 | |

**Notes.** BIC = Bayesian information criterion; AIC = Akaike information criterion; AvePP = Average posterior probability; OCC = Odds of correct classification.

^a^ For all models, the starting shape parameter for all trajectory groups was set to cubic.

^b^ Group AvePP of assignment: Based on the maximum probability assignment rule, individuals will be assigned to a group according to the largest posterior probability. For all the individuals assigned to a certain group, an AvePP will be calculated. For each trajectory group, an AvePP of assignment > 0.7 is indicative of good certainty of group assignments.

^c^ OCC: The numerator is the odds of a correct classification into a certain group based on the model, and the denominator is the correct classification into that group based on random assignment, essentially, OCC = [b/(1-b)]/[a/(1-a)]. For each trajectory group, OCC >= 5 suggests high assignment accuracy.

**Supplemental Table 11. Parameters of the best-fitting multi-trajectory model of frailty, cognitive impairment, and depressive symptoms (2011-2018; sensitivity analysis sample)**

|  | Parameter | Estimate | SE | t | P-value |
| --- | --- | --- | --- | --- | --- |
| Frailty | | | | | |
| Trajectory 1 | Intercept | 0.247 | 0.010 | 25.191 | 0.000 |
|  | Linear | -0.021 | 0.010 | -2.015 | 0.044 |
|  | Quadratic | 0.015 | 0.003 | 5.120 | 0.000 |
|  | Cubic | -0.001 | 0.000 | -5.495 | 0.000 |
| Trajectory 2 | Intercept | 0.169 | 0.008 | 21.538 | 0.000 |
|  | Linear | -0.010 | 0.008 | -1.208 | 0.227 |
|  | Quadratic | 0.008 | 0.002 | 3.403 | 0.001 |
|  | Cubic | -0.001 | 0.000 | -3.346 | 0.001 |
| Trajectory 3 | Intercept | 0.095 | 0.007 | 14.385 | 0.000 |
|  | Linear | -0.011 | 0.007 | -1.485 | 0.138 |
|  | Quadratic | 0.005 | 0.002 | 2.832 | 0.005 |
|  | Cubic | 0.000 | 0.000 | -2.534 | 0.011 |
| Trajectory 4 | Intercept | 0.054 | 0.006 | 9.211 | 0.000 |
|  | Linear | -0.006 | 0.006 | -0.998 | 0.318 |
|  | Quadratic | 0.004 | 0.002 | 2.368 | 0.018 |
|  | Cubic | 0.000 | 0.000 | -2.336 | 0.020 |
| Cognitive impairment | | | | | |
| Trajectory 1 | Intercept | 6.286 | 0.207 | 30.378 | 0.000 |
|  | Linear | -0.109 | 0.107 | -1.018 | 0.309 |
|  | Quadratic | -0.034 | 0.012 | -2.898 | 0.004 |
| Trajectory 2 | Intercept | 11.252 | 0.317 | 35.489 | 0.000 |
|  | Linear | 0.875 | 0.340 | 2.570 | 0.010 |
|  | Quadratic | -0.240 | 0.093 | -2.595 | 0.010 |
|  | Cubic | 0.014 | 0.007 | 2.077 | 0.038 |
| Trajectory 3 | Intercept | 6.480 | 0.141 | 46.100 | 0.000 |
|  | Linear | -0.017 | 0.073 | -0.228 | 0.819 |
|  | Quadratic | -0.049 | 0.008 | -6.277 | 0.000 |
| Trajectory 4 | Intercept | 12.956 | 0.122 | 106.009 | 0.000 |
|  | Linear | 0.114 | 0.064 | 1.797 | 0.072 |
|  | Quadratic | -0.035 | 0.007 | -5.048 | 0.000 |
| Depressive symptoms | | | | | |
| Trajectory 1 | Intercept | 18.053 | 0.747 | 24.177 | 0.000 |
|  | Linear | -4.678 | 0.804 | -5.817 | 0.000 |
|  | Quadratic | 1.289 | 0.219 | 5.883 | 0.000 |
|  | Cubic | -0.094 | 0.016 | -5.730 | 0.000 |
| Trajectory 2 | Intercept | 12.940 | 0.610 | 21.222 | 0.000 |
|  | Linear | -2.668 | 0.649 | -4.110 | 0.000 |
|  | Quadratic | 0.676 | 0.177 | 3.824 | 0.000 |
|  | Cubic | -0.046 | 0.013 | -3.450 | 0.001 |
| Trajectory 3 | Intercept | 10.657 | 0.510 | 20.888 | 0.000 |
|  | Linear | -2.278 | 0.549 | -4.154 | 0.000 |
|  | Quadratic | 0.535 | 0.150 | 3.578 | 0.000 |
|  | Cubic | -0.035 | 0.011 | -3.114 | 0.002 |
| Trajectory 4 | Intercept | 5.537 | 0.233 | 23.777 | 0.000 |
|  | Linear | -0.282 | 0.123 | -2.298 | 0.022 |
|  | Quadratic | 0.033 | 0.013 | 2.483 | 0.013 |

**Notes.** Trajectory 1 = Worsening frailty, worsening cognitive impairment, depression; Trajectory 2 = Declining pre-frailty, declining cognition, borderline depression; Trajectory 3 = Pre-frailty, worsening cognitive impairment, no depression; Trajectory 4 = Physically robust, declining cognition, no depression.

**Supplemental Table 12. Association between baseline characteristics and the trajectories of frailty, cognitive impairment, and depressive symptoms in Chinese older adults (2011-2018; sensitivity analysis sample)**

|  |  | **Trajectories of frailty, cognitive impairment, and depressive symptoms** | | | | | | | | |
| --- | --- | --- | --- | --- | --- | --- | --- | --- | --- | --- |
|  |  | (*Ref: Physically robust, declining cognition, no depression*) | | | | | | | | |
|  |  |  |  |  |  |  |  |  |  |  |
|  |  | Worsening frailty, worsening cognitive impairment, depression | | | Declining pre-frailty, declining cognition, borderline depression | | | Pre-frailty, worsening cognitive impairment, no depression | | |
|  | | aOR | 95% CI | | aOR | 95% CI | | aOR | 95% CI | |
| **Age** (every 5-year increase; continuous) | | 1.96* | (1.76- | 2.19) | 1.20* | (1.09- | 1.31) | 1.78* | (1.62- | 1.96) |
| **Female** (ref: Male) | | 2.84* | (2.22- | 3.65) | 1.69* | (1.41- | 2.03) | 2.31* | (1.90- | 2.82) |
| ***Human capital*** | |  |  |  |  |  |  |  |  |  |
| **Education** (ref: Elementary school or higher) | |  |  |  |  |  |  |  |  |  |
|  | No formal education | 16.70* | (12.26- | 22.76) | 1.42 | (1.08- | 1.88) | 26.82* | (20.89- | 34.44) |
|  | Less than elementary school | 2.83* | (2.07- | 3.86) | 1.46* | (1.18- | 1.79) | 4.48* | (3.55- | 5.66) |
| **Work status** (ref: Currently working) | |  |  |  |  |  |  |  |  |  |
|  | Retired | 2.34* | (1.80- | 3.05) | 1.45* | (1.19- | 1.77) | 1.08 | (0.86- | 1.34) |
|  | Not employed/Never worked | 1.88 | (0.91- | 3.88) | 1.35 | (0.76- | 2.39) | 0.80 | (0.40- | 1.57) |
| ***Social capital*** | |  |  |  |  |  |  |  |  |  |
| **Not married ^a^** (ref: Married) | | 1.29 | (0.97- | 1.73) | 1.24 | (0.98- | 1.57) | 1.30 | (1.02- | 1.66) |
| **Empty nester** (ref: Living with children) | | 0.84 | (0.67- | 1.06) | 0.97 | (0.81- | 1.15) | 0.82 | (0.68- | 0.99) |
| **No participation in any social activities** (ref: Any social activity participation) | | 1.97* | (1.56- | 2.48) | 1.20 | (1.01- | 1.42) | 1.63* | (1.36- | 1.96) |
| ***Financial capital*** | |  |  |  |  |  |  |  |  |  |
| **Childhood family financial situation ^b^** (ref: Same) | |  |  |  |  |  |  |  |  |  |
|  | A lot better off/somewhat better off | 0.97 | (0.59- | 1.58) | 1.21 | (0.89- | 1.63) | 1.30 | (0.93- | 1.83) |
|  | Somewhat worse off/A lot worse off | 1.63* | (1.28- | 2.06) | 1.27 | (1.06- | 1.53) | 1.17 | (0.96- | 1.42) |
| **Per capita household consumption** (ref: Quartile 4) | |  |  |  |  |  |  |  |  |  |
|  | Quartile 1 | 1.03 | (0.71- | 1.51) | 0.90 | (0.67- | 1.19) | 1.43 | (1.05- | 1.94) |
|  | Quartile 2 | 1.18 | (0.82- | 1.69) | 1.12 | (0.86- | 1.46) | 1.61* | (1.19- | 2.17) |
|  | Quartile 3 | 1.06 | (0.74- | 1.52) | 1.13 | (0.88- | 1.44) | 1.36 | (1.01- | 1.84) |
| ***Health capital*** | |  |  |  |  |  |  |  |  |  |
| **Childhood health ^c^** (ref: About average) | |  |  |  |  |  |  |  |  |  |
|  | Much healthier/somewhat healthier | 0.78 | (0.60- | 1.01) | 0.88 | (0.73- | 1.05) | 0.78 | (0.64- | 0.96) |
|  | Somewhat less healthy/Much less healthy | 1.50 | (1.07- | 2.10) | 1.48 | (1.12- | 1.95) | 1.11 | (0.82- | 1.50) |
| **Self-rated health** (ref: Fair) | |  |  |  |  |  |  |  |  |  |
|  | Very good/good | 0.27* | (0.18- | 0.41) | 0.30* | (0.23- | 0.39) | 0.60* | (0.48- | 0.75) |
|  | Poor/very poor | 10.58* | (8.14- | 13.76) | 4.74* | (3.89- | 5.77) | 2.35* | (1.86- | 2.96) |
| ***Structural factor*** | |  |  |  |  |  |  |  |  |  |
| ***Hukou* and residence** (ref: Urban *hukou*, urban residence) | |  |  |  |  |  |  |  |  |  |
|  | Rural *hukou*, rural residence | 3.79* | (2.59- | 5.57) | 1.39 | (1.09- | 1.78) | 4.46* | (3.23- | 6.15) |
|  | Rural *hukou*, urban residence | 2.22* | (1.46- | 3.38) | 0.96 | (0.72- | 1.27) | 2.87* | (2.01- | 4.09) |
|  | Urban *hukou*, rural residence | 1.71 | (0.78- | 3.73) | 1.10 | (0.70- | 1.74) | 1.77 | (0.95- | 3.29) |

**Notes.** Ref = reference. aOR = adjusted odds ratio. 95% CI = 95% confidence interval.

* P-value corrected for multiple comparisons (Holm step-down method) < 0.05. For specific P-values, both original and corrected for multiple comparisons, see Supplemental Table 13.

^a^ Included widowed, divorced, separated, and never married.

^b^ Self-rated family financial situation before age 17 years, compared to average family in the same community/village.

^c^ Self-rated health before age 16 years compared to other children of the same age.

**Supplemental Table 13. Association between baseline characteristics and the trajectories of frailty, cognitive impairment, and depressive symptoms in Chinese older adults (2011-2018; sensitivity analysis sample) ^a^: Original P-values and P-values corrected for multiple comparisons**

|  | | **Trajectories of frailty, cognitive impairment, and depressive symptoms** | | | | | |
| --- | --- | --- | --- | --- | --- | --- | --- |
|  | | (*Ref: Physically robust, declining cognition, no depression*) | | | | | |
|  | | Worsening frailty, worsening cognitive impairment, depression | | Declining pre-frailty, declining cognition, borderline depression | | Pre-frailty,  worsening cognitive impairment, no depression | |
|  | | Original ^b^ | Corrected ^c^ | Original ^b^ | Corrected ^c^ | Original ^b^ | Corrected ^c^ |
| **Age** (every 5-year increase; continuous) | | **0.000** | **0.000** | **0.000** | **0.000** | **0.000** | **0.000** |
| **Female** (ref: Male) | | **0.000** | **0.000** | **0.000** | **0.000** | **0.000** | **0.000** |
| ***Human capital*** | |  |  |  |  |  |  |
| **Education** (ref: Elementary school or higher) | |  |  |  |  |  |  |
|  | No formal education | **0.000** | **0.000** | **0.013** | 0.156 | **0.000** | **0.000** |
|  | Less than elementary school | **0.000** | **0.000** | **0.000** | **0.000** | **0.000** | **0.000** |
| **Work status** (ref: Currently working) | |  |  |  |  |  |  |
|  | Retired | **0.000** | **0.000** | **0.000** | **0.000** | 0.512 | 1.000 |
|  | Not employed/Never worked | 0.087 | 0.672 | 0.301 | 1.000 | 0.507 | 1.000 |
| ***Social capital*** | |  |  |  |  |  |  |
| **Not married ^a^** (ref: Married) | | 0.084 | 0.672 | 0.079 | 0.790 | **0.036** | 0.315 |
| **Empty nester** (ref: Living with children) | | 0.136 | 0.816 | 0.709 | 1.000 | **0.035** | 0.315 |
| **No participation in any social activities** (ref: Any social activity participation) | | **0.000** | **0.000** | **0.035** | 0.385 | **0.000** | **0.000** |
| ***Financial capital*** | |  |  |  |  |  |  |
| **Childhood family financial situation ^b^** (ref: Same) | |  |  |  |  |  |  |
|  | A lot better off/somewhat better off | 0.898 | 1.000 | 0.230 | 1.000 | 0.126 | 0.580 |
|  | Somewhat worse off/A lot worse off | **0.000** | **0.000** | **0.009** | 0.117 | 0.116 | 0.580 |
| **Per capita household consumption** (ref: Quartile 4) | |  |  |  |  |  |  |
|  | Quartile 1 | 0.861 | 1.000 | 0.445 | 1.000 | **0.024** | 0.240 |
|  | Quartile 2 | 0.386 | 1.000 | 0.394 | 1.000 | **0.002** | **0.024** |
|  | Quartile 3 | 0.752 | 1.000 | 0.342 | 1.000 | **0.045** | 0.315 |
| ***Health capital*** | |  |  |  |  |  |  |
| **Childhood health ^c^** (ref: About average) | |  |  |  |  |  |  |
|  | Much healthier/somewhat healthier | 0.055 | 0.495 | 0.159 | 1.000 | **0.016** | 0.176 |
|  | Somewhat less healthy/Much less healthy | **0.020** | 0.200 | **0.006** | 0.090 | 0.487 | 1.000 |
| **Self-rated health** (ref: Fair) | |  |  |  |  |  |  |
|  | Very good/good | **0.000** | **0.000** | **0.000** | **0.000** | **0.000** | **0.000** |
|  | Poor/very poor | **0.000** | **0.000** | **0.000** | **0.000** | **0.000** | **0.000** |
| ***Structural factor*** | |  |  |  |  |  |  |
| ***Hukou* and residence** (ref: Urban *hukou*, urban residence) | |  |  |  |  |  |  |
|  | Rural *hukou*, rural residence | **0.000** | **0.000** | **0.008** | 0.112 | **0.000** | **0.000** |
|  | Rural *hukou*, urban residence | **0.000** | **0.000** | 0.759 | 1.000 | **0.000** | **0.000** |
|  | Urban *hukou*, rural residence | 0.182 | 0.910 | 0.676 | 1.000 | 0.072 | 0.432 |

**Notes.**

^a^ Associations in adjusted odds ratios and 95% confidence intervals were summarized in Supplemental Table 12.

^b^ original P-values

^c^ P-values corrected by the Holm step-down method for multiple comparisons.
